# Combining ChIP-chip and Expression Profiling to Model the MoCRZ1 Mediated Circuit for Ca^2+^/Calcineurin Signaling in the Rice Blast Fungus

**DOI:** 10.1371/journal.ppat.1000909

**Published:** 2010-05-20

**Authors:** Soonok Kim, Jinnan Hu, Yeonyee Oh, Jongsun Park, Jinhee Choi, Yong-Hwan Lee, Ralph A. Dean, Thomas K. Mitchell

**Affiliations:** 1 Department of Plant Pathology, The Ohio State University, Columbus, Ohio, United States of America; 2 Center for Integrated Fungal Research, North Carolina State University, Raleigh, North Carolina, United States of America; 3 Department of Agricultural Biotechnology, Center for Agricultural Biomaterials, Center for Fungal Genetic Resources, and Center for Fungal Pathogenesis, Seoul National University, Seoul, Korea; University of Melbourne, Australia

## Abstract

Significant progress has been made in defining the central signaling networks in many organisms, but collectively we know little about the downstream targets of these networks and the genes they regulate. To reconstruct the regulatory circuit of calcineurin signal transduction via *MoCRZ1*, a *Magnaporthe oryzae* C2H2 transcription factor activated by calcineurin dephosphorylation, we used a combined approach of chromatin immunoprecipitation - chip (ChIP-chip), coupled with microarray expression studies. One hundred forty genes were identified as being both a direct target of MoCRZ1 and having expression concurrently differentially regulated in a calcium/calcineurin/MoCRZ1 dependent manner. Highly represented were genes involved in calcium signaling, small molecule transport, ion homeostasis, cell wall synthesis/maintenance, and fungal virulence. Of particular note, genes involved in vesicle mediated secretion necessary for establishing host associations, were also found. *MoCRZ1* itself was a target, suggesting a previously unreported autoregulation control point. The data also implicated a previously unreported feedback regulation mechanism of calcineurin activity. We propose that calcium/calcineurin regulated signal transduction circuits controlling development and pathogenicity manifest through multiple layers of regulation. We present results from the ChIP-chip and expression analysis along with a refined model of calcium/calcineurin signaling in this important plant pathogen.

## Author Summary

All organisms have the innate ability to perceive their environment and respond to it, largely through controlling gene expression. Tailored specificity of a response is primarily achieved through signal cascades involving unique receptors, downstream transcription factors (proteins that bind to DNA to regulate gene expression), and the genes these transcription factors regulate. For fungal plant pathogens, signal transduction cascades are involved in perception of hosts, transgression of physical barriers, suppression or elicitation of host defenses, *in vivo* nutrient acquisition, and completion of their life cycle. We know that the Ca^2+^/calcineurin signaling pathway is a central conduit regulating these aspects of the life cycle for fungal pathogens of plants and animals. In this study, we used advanced ChIP-chip and microarray gene expression technologies to identify the genes that the Ca^2+^/calcineurin responsive transcription factor MoCRZ1 directly binds to and regulates the expression of. Our findings show conservations and divergence in this pathway within the fungal kingdom. It also identifies points of control in the pathway that were previously unidentified. Most importantly, this study implicates this pathway in the establishment of host associations and virulence for the causal agent of rice blast disease, *Magnaporthe oryzae*, the most important disease of rice worldwide.

## Introduction

Rice blast, caused by the fungal pathogen *Magnaporthe oryzae*, is a recurrent and devastating problem worldwide [1]. Severe disease outbreaks can destroy upwards of 90% of rice yields for an entire field, region, or country resulting in a dramatic impact on human welfare and regional economies. The process starts when an asexual spore lands on a rice leaf. Given suitable moisture and temperature, the spore germinates with a short germination tube at the tip of which a specialized infection structure, an appressorium, emerges. The formation of an appressorium is essential for successful disease as it facilitates breaching the plant cuticle and cell wall, allowing access to the underlying tissues. Following penetration, the entry peg forms an un-branched hyphal strand that subsequently matures into branched bulbous infection hyphae. The fungus fills the infected cell in what is considered a biotrophic state before invading adjacent cells and switching to a necrotrophic state where it ramifies through the host tissues killing cells. In the final stage, the fungus produces new asexual spores that are spread to neighboring plants [2], [3]. While knowledge of the core signal pathways regulating each phase of this process continues to resolve, the key determinants controlling environmental perception and cellular response are as yet not fully understood [4]. Specifically, we have little knowledge of the upstream receptors used by the fungus to detect stimuli, nor do we know how downstream factors specifically interact to affect expression of genes deployed during infection related development, establishment of host associations, and invasive growth.

Calcium signaling has been implicated in regulating growth and development in *M. oryzae* including the infection process [5]–[9]. The components of Ca^2+^ signaling have been studied in many organisms and are relatively well understood. Ca^2+^ signaling starts when G-protein coupled receptors are activated by an external stimulus. Phospholipase C (PLC) is activated to hydrolyze phosphatidyl inositol-1,4-bisphosphate (PIP2) into inositol 1,4,5-triphosphate (IP3) and diacylglycerol. IP3 activates Ca^2+^ release from intracellular stores into the cytosol. Ca^2+^ ions bind to and activate calmodulin, which in turn, activates the Ca^2+^/calmodulin-dependent serine/threonine protein phosphatase calcineurin. Calcineurin is a heterodimer consisting of catalytic (CNA) and regulatory (CNB) subunits. In fungi, calcineurin mediated Ca^2+^ signaling has been shown to be required for growth, development, response to stress, and pathogenesis [10]. It was necessary for survival during environmental stresses such as ions (Mn^2+^, Li^+^, Na^+^), high pH, high temperature, ER stress, and prolonged incubation with mating pheromone α-factor in *Saccharomyces cerevisiae* [11], [12]. It is essential for growth and virulence of *Candida albicans* and *Cryptococcus neoformans* [13]–[16], and controls the dimorphic transition from mycelia to yeast in *Paracoccidioides basiliensis* [17]. Effects of gene deletion or chemical inhibition in filamentous fungi typically have pleiotropic effects. For example, a *cnaA* deletion mutant in *Aspergillus fumigatus* was viable but severely affected in hyphal morphology, sporulation, conidial architecture, pathogenicity, and invasive growth [18], [19]. Reduction of calcineurin activity by the immunosuppressant drug cyclosporine A, resulted in reduction of mycelial growth and alteration in hyphal morphology as shown in *Neurospora crassa* [20], [21], *A. nidulans* [22], *A. oryzae* [23], and *M. oryzae* [24]. RNA silencing in *M. oryzae* showed similar effects, specifically a reduction in mycelial growth, sporulation, and appressorium formation in *MCNA* knock down mutants [8].

Calcineurin functions mainly through the activation of the transcription factor CRZ1 (Calcineurin Responsive Zinc Finger 1). Upon activation by increased intracellular Ca^2+^ and calmodulin, calcineurin dephosphorylates CRZ1 leading to its nuclear localization. As a major mediator of calcineurin signaling, *crz*1 deletion mutants in a variety of fungi showed similar phenotypes as calcineurin mutants [5], [25]–[28]. However, differences have been noted. *CRZ1* in *C. albicans* was not involved in tolerance to antifungal agents (fluconazole, terbinafine) and only slightly affected in virulence, which is in contrast to the calcineurin mutants [28]. On the other hand, *CRZ1* is strongly associated with virulence both in human and plant pathogenic fungi [5], [25], [26], [28], [29]. The *B. cinerea CRZ1* ortholog *BcCRZ1* was required for growth, conidial and sclerotial development, and full virulence while being dispensable for conidia-derived infection of bean plants [25]. In *M. oryzae*, the *Δmocrz1* deletion mutant showed decreased conidiation and was not able to cause disease when spray inoculated. Mutant conidia were not distinguishable from that of wild type and formed appressoria at a similar level as wild type. Importantly, a significant portion of appressoria failed to penetrate rice sheath tissue. The mutant could colonize and cause disease when the conidia were infiltrated directly into the host tissue, thus bypassing the penetration process and suggesting that *MoCRZ1* plays a role in appressorium mediated penetration and establishing a biotrophic association with its host [5].

Comprehensive genome-wide expression analysis in *S. cerevisiae* identified 163 genes regulated in a calcineurin/CRZ1 dependent manner by the stimulation of Ca^2+^ or Na^+^. These genes were associated with a diverse range of cellular processes including signaling pathways, ion/small molecule transport, cell wall synthesis/maintenance, and vesicular trafficking [30]. In *C. albicans*, microarray analysis revealed 60 genes to be transcriptionally activated by exogenous Ca^2+^ treatment through calcineurin/CRZ1 regulation using *cnaΔ/Δ* and *crz1Δ/Δ* mutants. Analysis of putative functions revealed that about 60% of these genes were involved in cell wall organization, cellular organization, cellular transport and homeostasis, cell metabolism, and protein fate [28]. Many of the genes regulated through calcineurin signaling in these two species belonged to similar functional groups, although only 9 genes were found to be commonly regulated [28]. To date, no genome-wide study has been conducted to identify regulated genes by direct binding of CRZ1 to promoter regions.

Here we report the use of chromatin immunoprecipitation coupled with non-coding region tiling arrays (ChIP-chip) analysis and whole-genome expression studies to identify target genes directly regulated by MoCRZ1. To our knowledge, this is the first report of ChIP-chip technology being applied to filamentous fungi. From this analysis, we can model the Ca^2+^/calcineurin signaling and control pathways that in-part influence infection related development and establishment of a compatible host association for this devastating plant pathogen. Further, this work reveals divergence within the fungal kingdom of the suites of genes and processes directly regulated by this ubiquitous signaling pathway.

## Results

### GFP tagging of MoCRZ1 and localization

**Figure 1:**
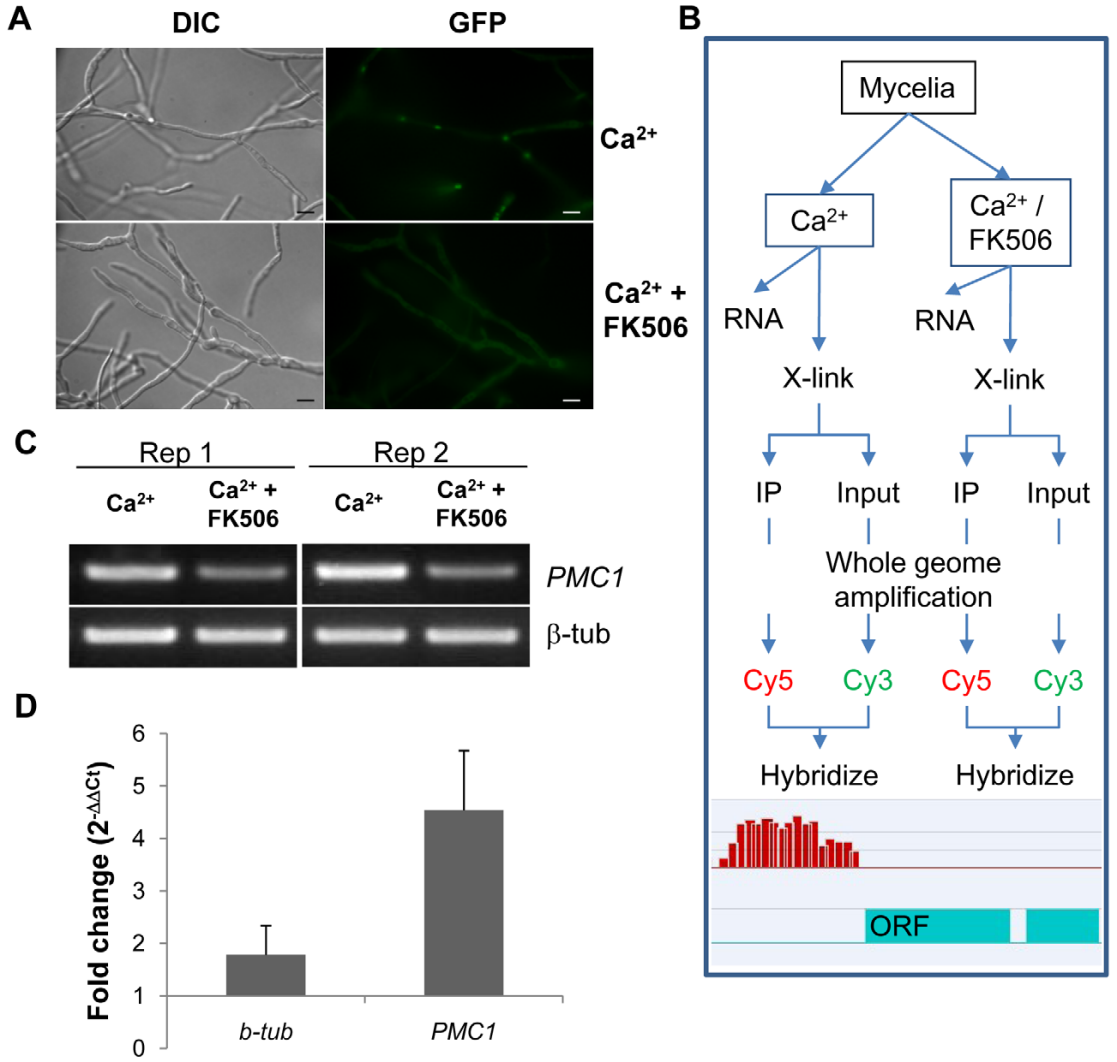
Establishment of Chromatin immunoprecipitation. (A) Nuclear localization of MoCRZ1 was visualized in the eGFP tagged strain under the native promoter. FK506 blocks nuclear localization of MoCRZ1::eGFP. Bar indicates 10 µm. (B) ChIP-chip experimental design to identify MoCRZ1 targets activated by calcium treatment. The Ca^2+^/FK506 treated sample served as the negative control treatment. (C) RT-PCR to verify that *PMC1* was up-regulated in the Ca^2+^ treated but not in Ca^2+^/FK506 treated sample. (D) Quantitative PCR was conducted with DNA after ChIP with antiGFP antibody. 30% input DNA collected prior to pull down was used as control. 1 µl each of ChIPed and input DNA was used for real-time PCR. Fold changes were calculated by 2^ΔΔCt^, where ΔΔCt = (Ct_input DNA_-Ct_ChIPed DNA_) _Ca2+ treated sample_ - (Ct_input DNA_-Ct_ChIPed DNA_) _Ca2+/FK506 treated sample_.

A MoCRZ1::eGFP construct was co-transformed into fungal protoplasts along with a hygromycin resistance conferring vector. Transformants were single spore isolated and screened under the epi-fluorescent microscope. MoCRZ1-eGFP fluorescence was faint and evenly dispersed through the cytosol in mycelia with nuclear localization detected in hyphal tips (data not shown). Nuclear translocation of CRZ1 in response to Ca^2+^ is a well conserved phenomenon and was shown previously to occur in *M. oryzae* [5]. Following mycelia treatment with CaCl_2_, eGFP fluorescence was localized to the nucleus (Figure 1A), as expected. Addition of the calcineurin inhibitor FK506 completely blocked nuclear accumulation of the fluorescent protein. The MoCRZ1::eGFP over-expression line showed normal growth, appressoria development, and virulence to susceptible rice cultivar Nipponbare (data not shown), and was selected for subsequent ChIP-chip analysis.

### Chromatin immunoprecipitation of MoCRZ1 bound sequences

Experimental design for ChIP-chip analysis is depicted in Figure 1B. CaCl_2_ treated mycelia were used to enrich MoCRZ1 occupied genomic fragments, while mycelia treated with CaCl_2_ and FK506 were used as the negative control. Expression of *PMC1* (P-type ATPase) was analyzed for each sample by RT-PCR as shown in Figure 1C to confirm the effect of each treatment, i.e., up-regulated in the calcium treated mycelium and blocked by FK506. *PMC1* is a previously described target of MoCRZ1 [5] and was used throughout this study as positive control marker. Enrichment of MoCRZ1 bound DNA fragments in ChIPed fractions when compared to input DNA (non-ChIPed) was confirmed by real-time PCR (Figure 1D). As expected, the fold change of *PMC1* was 4.54±1.13 in ChIPed DNA from Ca^2+^ treated mycelia over input DNA from Ca^2+^ + FK506 treated mycelia, while that of β-tubulin was 1.78±0.56 (Figure 1D). Input and IPed DNA was amplified and subsequently re-amplified to amass sufficient DNA (∼8.5 ug) for labeling and hybridization to the array. Enrichment of MoCRZ1 bound DNA in the ChIPed fraction was validated by PCR amplification of *PMC1* at each step (data not shown). ChIPed DNA labeled with Cy5 was co-hybridized with Cy3 labeled input DNA to the NimbleGen *M. oryzae* intergenic region specific tiling array (see Materials and Methods for array description).

### Genome-wide identification of MoCRZ1 binding sites and downstream genes

**Figure 2:**
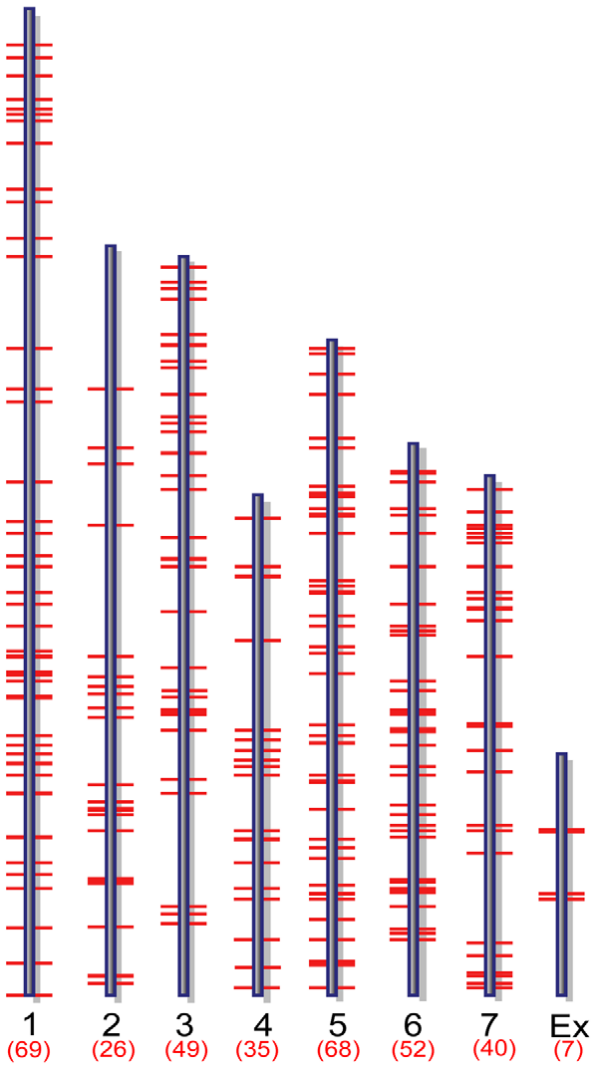
Genome-wide distribution of putative MoCRZ1 targets. Target genes mapped to *M. oryzae* supercontigs version 5.

Two complementary approaches were applied to analyze ChIP-chip hybridization data to identify putative MoCRZ1 binding sites and the genes it regulates. Initially, 42 peaks were identified as having a false discovery rate less than 0.2 and being common in both biological replications of the Ca^2+^ treatment but not in the Ca^2+^+FK506 treatment. The 42 peaks were within 1 kb upstream of 37 predicted genes. Following this initial analysis, we used NimbleGen's SignalMap software to manually interrogate the ratio signal tracks across the genome to identify short sequence stretches showing a normal distribution profile of signal intensities upstream of annotated ORFs (Figure 1B bottom). Sequence tracks showing this profile were accepted only if they appeared in both biological replications of the Ca^2+^ treatment with no or lower signal intensities in the Ca^2+^+FK506 treatment. If the binding signals were located between two divergently transcribed ORFs, both ORFs were regarded as possible candidates. This manual analysis resulted in the identification of 346 genes evenly distributed through the genome with no apparent bias (Figure 2, Table S1). Importantly, the 37 genes resulting from the first automated analysis were captured in the set of 346. Manual analysis produced more putative targets than the automated because SignalMap reports the probe ID with the highest signal intensity. In most cases, a single binding site did not share the identical probe as having the highest signal between biological replicates, thereby disqualifying them from the automated analysis. Data was deposited in the Gene Expression Omnibus (GEO) at NCBI under the accession number of GSE18180 (http://www.ncbi.nlm.nih.gov/geo/query/acc.cgi?token=dlarvwwmsoquoxs&acc=GSE18080).

### Microarray validation of ChIP-chip results

**Figure 3:**
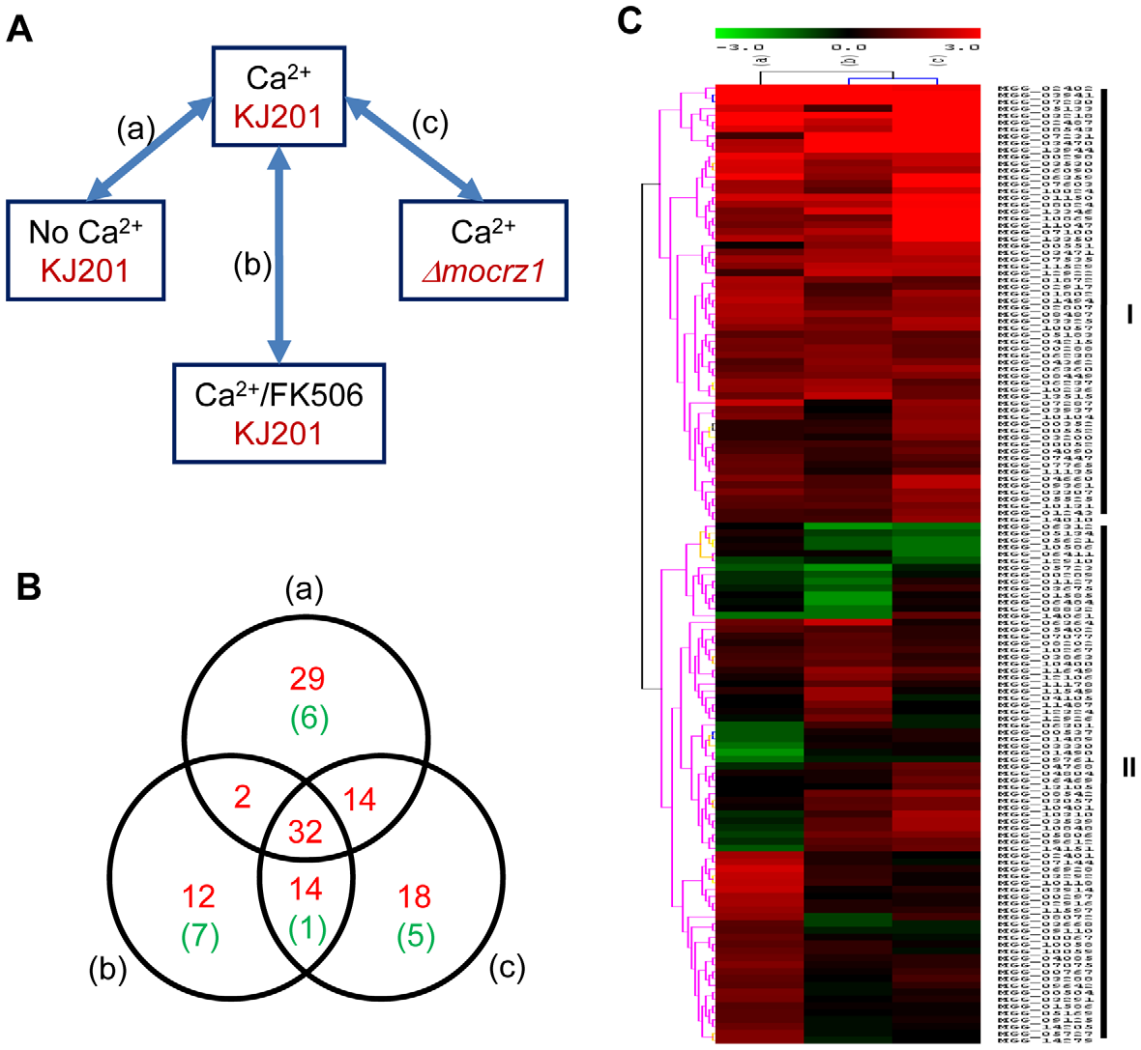
Expression dynamics of MoCRZ1 targets. (A) Expression microarray design. Wild type strain KJ201 and *MoCRZ1* deletion mutant (*Δmocrz1*) were treated with Ca^2+^ and/or FK506 as depicted. Agilent *M. oryzae* whole genome microarray chip ver. 2 was hybridized in a single channel design with four biological replications per treatment. After global normalization of signal intensities to the average expression level of all probes among the 16 data sets, pairwise comparison between treatments was conducted. (B) Venn diagram showing number of genes identified from ChIP-chip and up-regulated in transcriptome profiling described in panel A. Number of genes with more than 2 fold differential expression with *P*<0.05 were noted as red for up-regulation and green for down-regulation in Ca^2+^ treated wild type samples in each comparison. (C) Hierachical clustering resulted in two large groups with differential expression.

Expression microarray analysis was conducted to corroborate genes predicted by ChIP-chip to be regulated by MoCRZ1. The experimental design is described in Figure 3A. Wild type strain KJ201 was treated with CaCl_2_ without or with FK506 to identify calcium and calcineurin dependent genes, respectively. *MoCRZ1* deletion mutant (*Δmocrz1*) was also treated with CaCl_2_ to identify *MoCRZ1* regulated genes. Four biological replicates for each of the 4 treatments were selected for hybridization to the Agilent *M. oryzae* whole genome microarray chip version 2. Signal intensities from the single channel hybridization were normalized to the average expression level of all probes among the 16 data sets. Pair wise comparison between treatments was conducted as depicted in Figure 3A, in which (a), compared Ca^2+^ treated/no treatment in wild type strain KJ201 (CA/CK); (b), Ca^2+^ treated/Ca^2+^+FK506 treated in KJ201 (CA/CAFK); (c), Ca^2+^ treatments in KJ201/Ca^2+^ treatments in *Δmocrz1* (CA/CRZ). Genes were regarded as differentially expressed if their average signal intensity among 4 replicates was above 20 in a minimum of one condition and the expression ratio is greater than 2 fold with *P*<0.05 (Student's t-test). Changes in gene expression of the 346 genes identified from ChIP-chip were analyzed. Of the 346, we found 309 with expression in each condition, with 121 and 19 genes up- or down- regulated, respectively, in the Ca^2+^ treated KJ201 condition in at least one comparison (Table S1, Figure 3B). It was noteworthy that the expression level of some MoCRZ1 target genes was lower in the Ca^2+^ activated wild type condition than in calcineurin and/or *MoCRZ1* defective conditions suggesting that MoCRZ1 can act as repressor. These 140 (121+19) genes represent those directly bound to and regulated by MoCRZ1, and form the set used for analyses described below. The full dataset was deposited in NCBI GEO with the accession number of GSE18185 (http://www.ncbi.nlm.nih.gov/geo/query/acc.cgi?token=ttmdlgumsmsoury&acc=GSE18185). SuperSeries GSE18193 combining the ChIP-chip and microarray data were also generated (http://www.ncbi.nlm.nih.gov/geo/query/acc.cgi?token=tnutnsemyikamlm&acc=GSE18193).

### Validation of gene expression by real-time RT-PCR

**Figure 4:**
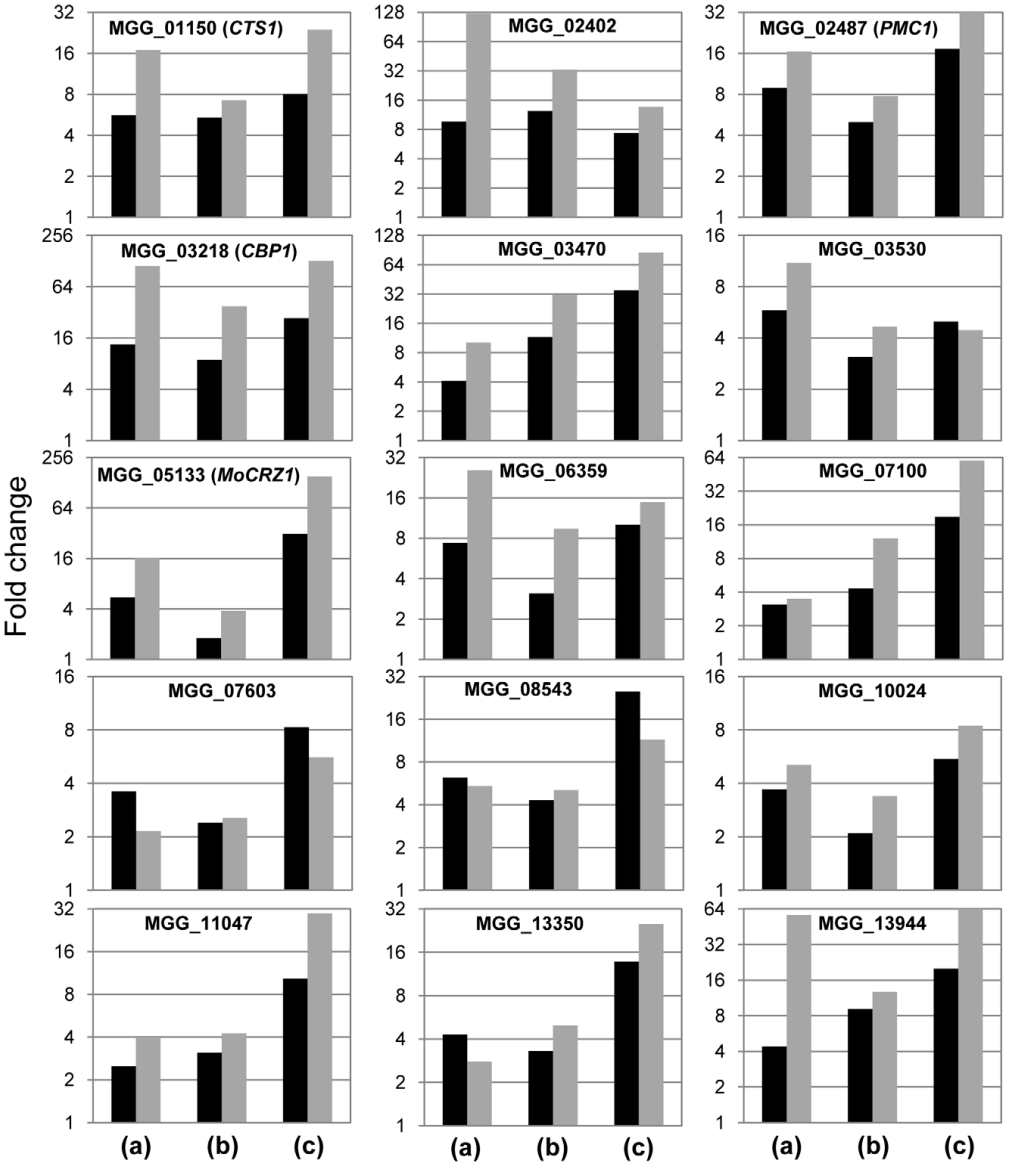
Real-time RT-PCR to validate differential expression. cDNA relevant to 25 ng total RNA was used to run real-time RT-PCR. Log_2_^(Fold changes)^ calculated by 2^−ΔΔCt^ were displayed. ΔΔCt = (Ct_gene_ _of interest_-Ct_control gene_)_test condition_-(Ct_gene_ _of interest_-Ct_control gene_)_control condition_. Black bar represents signal ratio from microarray data, while grey bar shows fold changes from real-time RT-PCR. Fold changes were calculated by normalizing Ct values as in Figure 3A, where (a) compares expression level between Ca^2+^ treated vs. no treatment in wild type strain KJ201; (b), Ca^2+^ vs. Ca^2+^+FK506 in KJ201; (c), Ca^2+^ treatments in KJ201 vs. in *Δmocrz1*.

The 15 most up-regulated genes in calcium treated wild type strain KJ201compared to that of the *Δmocrz1* mutant were selected for real-time RT-PCR to validate expression data. The results in Figure 4 show that each gene is transcriptionally more activated in all three comparison, i.e., Ca^2+^ treated vs. untreated control (a), Ca^2+^ treated vs. Ca^2+^ + FK506 treatment (b), and Ca^2+^ treated wild type vs. *Δmocrz1* (c). Although the magnitude of fold changes was much higher than that from microarray in most cases, real-time RT-PCR data supported the microarray results.

### Identification of novel and known MoCRZ1 regulated genes

Functional categorization was conducted in two ways. At first, hierarchical classification according to the expression pattern resulted in two groups. This analysis was followed by GO annotation using an InterPro to GO module incorporated in the Comparative Functional Genomics Platform (Figure 3C and Table S2, S3) [31]. Group I contains 64 genes of which expression was tightly regulated in a Ca^2+^/calcineurin/*MoCRZ1* dependent fasion, i.e., up-regulated in all three comparisons. Group II comprises 76 genes whose expression was differentially regulated in three comparisons. Twenty four genes of group I and 36 of group II were assigned with GO terms, 14 and 22 to biological process, 20 and 33 to molecular function, 10 and 19 to cellular component, respectively (Table S2). Second, genes were annotated through literature with their specific functions assigned according to the functional classes of Cyert [32] (Table 1). Sixty-two of 140 genes identified by both ChIP-chip and microarray analyses could be assigned to one of 7 functional groups (Table 1). Consistent with the role of MoCRZ1 in providing tolerance to ionic and cell wall stress, genes involved in small molecule transport or ion homeostasis and cell wall synthesis/maintenance were highly represented. Among them was *PMC1*, which provides support for the validity of these results. Furthermore, the AAA family of ATPase as a whole was highly represented as direct targets of MoCRZ1, as well as members of major facilitator superfamily of multidrug-resistance proteins. Considering cell wall synthesis/maintenance genes, a number of GPI-anchored cell surface glycoproteins were captured in addition to the previously known downstream genes like chitin synthase activator (Chs3) and chitin syntase 1. Small secretory proteins, including effectors and cell wall degrading proteins, are regarded as key molecules acting at the interface between the plant and microbe. Efficient secretion of these proteins is assumed to be essential during the interaction between host and pathogen. Among the targets identified were genes comprising the vesicle mediated secretory pathway, including rhomboid family membrane protein (MGG_07535), Sso1/2 type SNARE protein (MGG_04090) known to be localized at secretory vesicles from Golgi to plasmamembrane, homocysteine S-methyltransferase (MGG_04215), golgi apyrase (MGG_07077), and a protein required for assembly of ER-to-Golgi SNARE complex (MGG_01489). Proper protein folding in the ER mediated by co-chaperone *LHS1* [33], and efficient Golgi performance involving exocytosis entailing functions of the integral membrane P-type ATPase encoded by *MgAPT2* [34], have been reported to be necessary for protein secretion and biotrophic phase infection in this fungus.

**Table 1:** Functional classification of MoCRZ1 direct targets.

Gene_ID	Description	Expression level in microarray^a^					
		CA/CK	*P* b	CA/CAFK	*P*	CA/CRZ	*P*
Signaling							
MGG_03218	calcineurin binding protein	13.49	0.002	8.88	0.001	27.31	0.002
MGG_06928	serine/threonine-protein kinase bur-1	5.60	0.002	–		–	
MGG_01150	calcineurin temperature suppressor Cts1	5.55	0.010	5.40	0.012	8.00	0.007
MGG_07287	lysophospholipase 3	4.92	0.005	–		3.29	0.002
MGG_04660	negative regulator of the PHO system	2.84	0.001	1.84	0.027	4.56	0.000
MGG_03937	serine/threonine protein kinase	2.52	0.018	–		3.08	0.001
MGG_06360	Annexin	2.10	0.018	2.88	0.004	3.61	0.002
MGG_03941	von Willebrand factor typeA (adhesion)	–		40.34	0.043	20.53	0.021
MGG_00552	acid phosphatase	–		1.48	0.046	3.33	0.001
MGG_11649	PX domain-containing protein	–		3.35	0.001	2.18	0.004
MGG_11178	Rho guanyl nucleotide exchange factor	–		2.10	0.001	–	
Transcription factor							
MGG_05133	MoCRZ1, C2H2 Transcription factor	5.51	0.006	–		31.37	0.029
MGG_13350	C6 zinc finger domain protein	4.34	0.005	3.27	0.010	13.78	0.002
MGG_02916	Transcriptional repressor	3.88	0.004	–		–	
MGG_02401	HIT finger domain protein	3.84	0.007	–		–	
MGG_00504	zinc finger protein 740	2.70	0.010	–		–	
MGG_06364	ACE1_TRIRE Zinc-finger transcription factor ace1	2.44	0.014	5.08	0.024	–	
MGG_03288	bZIP transcription factor	2.24	0.002	–		–	
MGG_04362	LPS induced transcription factor, LITAF-like zinc finger protein	1.95	0.005	3.13	0.004	2.67	0.001
MGG_01490	Transcriptional regulator, AraC family	0.29	0.024	–		–	
MGG_01127	C2H2 transcription factor (Rpn4)	–		0.38	0.010	–	
MGG_06312	C6 zinc finger domain-containing protein	–		0.27	0.001	0.43	0.010
MGG_12926	PHD and RING finger domain protein	–		2.61	0.014	–	
Ion, small molecule transport							
MGG_02487	Ca^2+^ transporting ATPase, PMC1	8.95	0.002	5.00	0.001	17.20	0.000
MGG_10118	ATPase family AAA domain-containing protein 1	4.74	0.007	–		–	
MGG_07075	ATPase family AAA domain-containing protein 1-A	2.91	0.010	–		–	
MGG_10869	MFS drug efflux transporter	2.78	0.041	–		15.94	0.004
MGG_04085	AAA family ATPase	2.49	0.001	–		1.40	0.012
MGG_01586	arsenite resistance protein Ars2	1.99	0.011	–		–	
MGG_00537	ammonium transporter MEP1	0.47	0.000	–		–	
MGG_00289	amino-acid permease inda1	–		0.46	0.049	–	
MGG_05723	fluconazole resistance protein 1	–		0.25	0.011	–	
MGG_06469	potassium transport protein 1	–		–		2.38	0.000
MGG_10131	major facilitator superfamily multidrug-resistance protein	–		2.15	0.027	2.73	0.009
MGG_12922	phospholipid-transporting ATPase 1	–		5.01	0.005	4.39	0.002
Vesicle trafficking							
MGG_07535	rhomboid family membrane protein	3.59	0.005	3.76	0.001	4.14	0.001
MGG_04215	homocysteine S-methyltransferase	2.62	0.003	2.36	0.004	2.22	0.004
MGG_03668	Importin subunit beta-1	2.14	0.006	–		–	
MGG_04090	SNARE protein	1.80	0.016	–		2.37	0.000
MGG_07077	golgi apyrase	1.51	0.041	2.11	0.008	–	
MGG_01489	required for assembly of the ER-to-Golgi SNARE complex	0.48	0.003	–			
Cell wall related							
MGG_07230	alpha-1,3-mannosyltransferase CMT1	10.48	0.026	24.12	0.022	17.37	0.002
MGG_03530	chitin synthase activator (Chs3)	5.81	0.012	3.08	0.011	4.99	0.004
MGG_01494	GPI-anchored cell surface glycoprotein	4.57	0.022	2.19	0.045	3.08	0.015
MGG_01802	chitin synthase 1	4.15	0.003	1.81	0.040	3.62	0.002
MGG_01872	putative cell wall protein	4.01	0.012	2.61	0.011	2.05	0.022
MGG_02917	GPI-anchored cell surface glycoprotein	3.79	0.009	–		2.19	0.028
MGG_03307	glutamine-serine-proline rich protein	2.66	0.002	2.03	0.004	3.13	0.001
MGG_07447	Anchorage subunit of a-agglutinin of a-cells	2.16	0.003	1.47	0.031	2.04	0.001
MGG_10058	GPI-anchored cell surface glycoprotein	2.05	0.031	–		–	
MGG_06301	GPI-anchored cell surface glycoprotein	0.46	0.027	1.72	0.020	–	
MGG_00352	GPI-anchored cell surface glycoprotein	–		–		3.33	0.001
MGG_00551	laccase-3	–		2.99	0.000	5.06	0.000
MGG_03539	GPI-anchored cell surface glycoprotein	–		–		3.73	0.005
MGG_08487	cellobiose dehydrogenase	–		3.33	0.034	3.23	0.027
MGG_10400	glucan 1,3-beta-glucosidase	–		2.13	0.014	–	
Degradative enzymes							
MGG_10104	zinc metalloprotease mde10 precursor	2.79	0.005	–		2.96	0.001
MGG_03200	zinc metalloprotease mde10 precursor	–		–		2.92	0.000
MGG_07765	F-box domain protein	2.31	0.031	–		–	
Lipid, sterol synthesis							
MGG_08072	cholesterol oxidase	3.17	0.004	0.64	0.037	1.67	0.025
MGG_08202	Cyclopropane-fatty-acyl-phospholipid synthase	–		1.97	0.000	1.49	0.011
MGG_08832	C-5 sterol desaturase	–		0.37	0.037	–	

a.Signal ratio between two treatments was calculated. CA/CK, Ca^2+^ treated/no treatment in wild type strain KJ201; CA/CAFK, Ca^2+^ treated/Ca^2+^+FK506 treated in KJ201; CA/CRZ, Ca^2+^ treatments in KJ201 vs. in *Δmocrz1*. Data with *P*<0.05 was described. – means signal ratio with *P* value higher than P≥0.05.

b.*P* value of the Student's t-test between to conditions.

A major group of genes found in this study to be regulated by MoCRZ1 are those involved in cellular signaling and transcription. Among them were genes encoding serine/threonine protein kinases (MGG_04660, MGG_07287, MGG_06928), a phosphatase (MGG_00552), and a Rho guanyl nucleotide exchange factor (MGG_11178). In addition, genes comprising calcium signaling machinery were also common. Genes encoding annexin (MGG_06360), lysophospholipase 3 (MGG_07287), PX domain-containing protein (MGG_11649), calcineurin binding protein (*CBP1*; MGG_03218) and the calcineurin temperature suppressor *CTS1* (MGG_01150) were identified. Of note, the expression of *CBP1* and *CTS1* was highly increased in all three comparisons. Binding in the promoter of these two genes and regulation of their transcription strongly suggests a previously uncharacterized level of negative feedback regulating calcineurin activity. MoCRZ1 functions to activate 12 genes from diverse families of transcription factors. Significantly, MoCRZ1 itself was identified, suggesting an autoregulatory role not previously reported. The expression of MoCRZ1 was induced by exogenous calcium treatment, but not altered by the inhibition of calcineurin activity with FK506. Regarding the fact that calcineurin dephosphorylates MoCRZ1 upon activation by calcium and that FK506 blocks nuclear localization of MoCRZ1, inactivation of calcineurin regulates function only at post-translational level. This data suggests an additional level of regulation at transcription.

### Classification of pathogenicity related genes regulated by MoCRZ1

*Δmocrz1* mutants are defective in post appressoria formation penetration and establishment of biotrophic host association. Appressoria from this mutant background have defects in penetration, however those that successfully penetrated fail to incite disease. We examined MoCRZ1 target genes for previously defined roles in fungal virulence. Of the 140 target genes queried to pathogen-host interactions database (PHI-base) version 3.1 [35], [36], 16 had matches to more than one entry from plant or human pathogens using a stringent cut-off value e<−20 (Table 2). Three proteins, *MoCRZ1* itself [5], MGG_03530 encoding chitin syntase activator Chs3 [37] and *PMC1* [8] were previously characterized to regulate virulence in *M. oryzae*. Similarly, members of ATPases family, *PDE1* [38] and *MgAPT2* [34], are known to be involved in *M. oryzae* pathogenicity. Two genes encoding serine/threonine protein kinases (MGG_04660 and MGG_06928) had 45 and 27 hits, respectively. Genes involved in drug resistance (MGG_05723 and MGG_10869), MGG_07230 alpha-1,3-mannosyltransferase CMT1, MGG_07287 lysophospholipase 3, MGG_05727 ankyrin repeat protein, MGG_03288 bZIP transcription factor, and MGG_09361 homolog of CgDN3 were similarly listed as being involved in fungal virulence.

**Table 2:** Genes implicated in fungal virulence.

Gene ID	Number of hits	PHI entries from^a^	Gene	Species^b^
MGG_00551 laccase-3	2	B	Lac2	Cn, Bc
MGG_01802 chitin synthase 1	7	B	BcCHS3a	Bc, Af, Wd, Fo, Ca
MGG_03530 chitin synthase activator (Chs3)	1	P	MGG_03530	Mo
MGG_02487 Ca transporting ATPase PMC1	1	H	PMC1	Ca
MGG_07230 alpha-1,3-mannosyltransferase CMT1	1	H	CAP59	Cn
MGG_07287 lysophospholipase 3	2	H	PLB1	Ca, Cn
MGG_12922 phospholipid-transporting ATPase 1	1	P	PDE1	Mo
MGG_07075 AAA type ATPase family	2	P	PEX6	Mo, Cl
MGG_10118 AAA type ATPase family	2	P	PEX6	Mo, Cl
MGG_03288 bZIP transcription factor	1	P	ZIF	Fg
MGG_05133 *MoCRZ1* C2H2 transcription factor		B	CRZ1	Af, Ca, Bc
MGG_05723 fluconazole resistance protein 1	2	B	CTB4	Ck, Ca
MGG_10869 MFS drug efflux transporter	3	P	BcMFS1	Bc, Cc, Ck
MGG_04660 negative regulator of the PHO system	45	B		Ca, Mo, etc^c^
MGG_06928 serine/threonine-protein kinase bur-1	27	B		Ca, Fg, etc^c^
MGG_05727 ankyrin repeat protein	1	H	KER1	Ca
MGG_09361 hypothetical protein	1	P	CgDN3	Cg

a Genes were characterized in human pathogen (H), plant pathogen (P), or both (B).

b Fungal species were abbreviated. Af; *Aspergillus fumigates*; Bc, *Botrytis cinerea*; Ca, *Candida albicans*; Cc, *Cochliobolus carbonum*; Cg, *Colletotrichum gloeosporioides*; Ck, *Cercospora kikuchii*; Cl, *Colletotrichum lagenarium*; Cn, *Cryptococcus neoformans*; Fg, *Fusarium graminearum*; Fo, *Fusarium oxysporum*; Mo, *Magnaporthe oryzae*; Wd, *Wangiella dermatitidis*.

c Fungal species of top 2 hits were listed.

### Analysis of the MoCRZ1 binding motif

**Figure 5:**
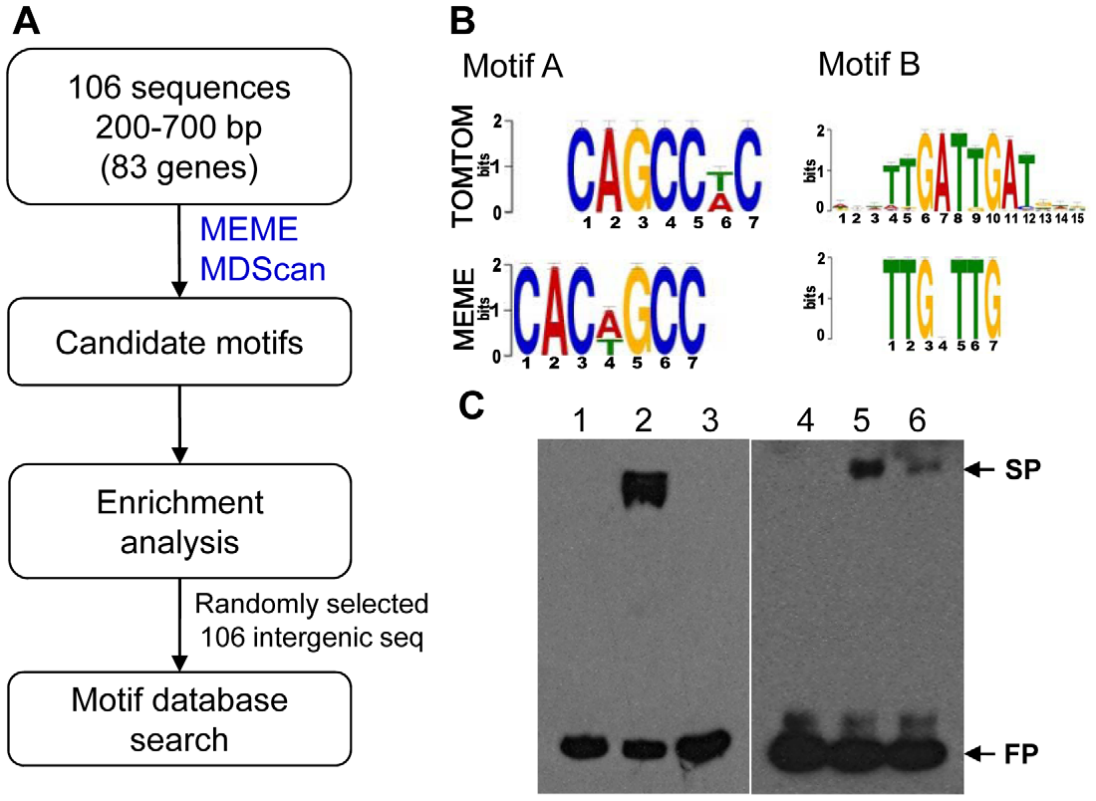
Putative MoCRZ1 binding motifs identified. (A) Analysis schema to identify putative MoCRZ1 binding motifs. (B) WebLogo of Top 2 motifs and the best hits in the yeast motif database. The consensus sequences of the putative motif sequences (MEME) calculated using WebLogo server were displayed in order of predominance from top to bottom at each position with large letters being higher frequency. Best hit from yeast motif database was displayed for comparison (TOMTOM). (C) Electrophoretic mobility shift assay. Probe DNA was amplified by PCR from the promoter regions of *MoCRZ1* (left panel, lanes 1 to 3) and *CBP1* (right panel, lanes 4 to 6) with 5′-Biotin-labeled primer pairs, purified by gel isolation, and allowed to bind to purified MoCRZ1 protein. Lanes 1 and 4, Biotin-labeled probe DNA; lanes 2 and 5, probe DNA with purified MoCRZ1 protein; lanes 3 and 6, competition reaction with 200 fold molar excess of unlabeded DNA. FP, free probe; SP, shifted probe. Reaction mixture was run on 5% polyacrylamide gel in 0.5X TBE and transferred onto Hybond N^+^ (GE Healthcare) membrane. Signals were detected using chemiluminescence.

To identify the MoCRZ1 binding motif, the exact binding sequences of MoCRZ1 (∼50–1247 bp) revealed from ChIP-chip studies were retrieved from the promoters of genes in common between ChIP-chip and microarray expression studies and subjected to motif signature analyses (Figure 5A). Initially, 106 sequences from each of the 83 genes differentially regulated in the WT/*Δmocrz1* comparison (Figure 3B) were analyzed with MEME [39] and MDScan [40] (Figure 5A). There were more sequences than genes as 21 genes had 2 peaks in their promoter region and 2 genes had 3. Candidate motifs from both algorithms were manually interrogated and enumerated to identify the top 3 candidates, which were subsequently screened against randomly retrieved 106 intergenic sequences with an average length of 509 bp (Figure 5A). The most enriched motif of CAC[AT]GCC was identified in 33 sequences in front of 24 genes, a 16X enrichment in MoCRZ1 bound sequences. The most common motif of TTGNTTG was found in 68 promoter sequences in front of 42 genes with 4X enrichment. Motif TAC[AC]GTA occurred in 22 promoter sequences of 18 genes with 4X enrichment. Fifty-six genes had at least one motif, while all three of these motifs occurred in front of 5 genes including *PMC1*, *CTS1*, MGG_01494 encoding a cell wall protein, and two genes encoding conserved hypothetical proteins MGG_03539 and MGG_06359 (Table S4). These motifs were searched against yeast motif database using TOMTOM [41]. The top match for CAC[AT]GCC was MET28 (*p*-value = 0.0013), while the second match was CRZ1 with significant *p*-value (0.0022), showing Crz1p of *S. cerevisiae* has this motif in its promoters although it was not previously identified as a calcineurin dependent response element (CDRE) (Figure 5B). Pbx1b (*p*-value = 0.00039) and Zec (*p*-value = 0.00045) were best two matches for TTGNTTG, while no significant match was returned for TAC[AC]GTA. Binding of MoCRZ1 to the promoter region was confirmed by Electrophoretic Mobility Shift Assay (EMSA). A 209 bp PCR fragment having 1 TTGNTTG and 2 CAC[AT]GCC motifs from the *MoCRZ1* promoter region was bound to purified MoCRZ1 protein (Figure 5C, left panel). A 325 bp fragment of *CBP1* (MGG_03218) having 1 TTGNTTG and 1 CAC[AT]GCC motifs was also shown to bind to purified MoCRZ1 (Figure 5C, right panel).

## Discussion

The most common signal transduction pathways in nature, MAPK, cAMP, and calcium mediated, have been shown to be involved in all aspects of growth, development, and pathogenicity of *M. oryzae* and several other fungal pathogens of plants. Recently, a transcription factor, *MoCRZ1*, relaying calcium signals from calcineurin has been characterized [5], [42]. As a major mediator of calcineurin signaling, *crz1* deletion mutants in a variety of fungi showed similar phenotypes as calcineurin mutants such as sensitivity to Ca^2+^ and other ionic stresses as well as cell wall stresses. In addition, *Δmocrz1* showed defects in development and pathogenicity, including reduced conidiation and defective appressorium mediated penetration [5], [42]. *MoCRZ1* is required for the calcineurin-dependent transcriptional induction of downstream genes such as *PMC1*, encoding P-type ATPase, *CHS2* and *CHS4*, encoding chitin synthase. In this study, we gained a genome-wide perspective of the direct downstream targets of *MoCRZ1* signaling using a combined approach of ChIP-chip and expression microarray analysis. CRZ1 in filamentous fungi have common as well as unique roles compared to those of their yeast ortholog. As such, we find that the suite of genes regulated by MoCRZ1 contains a high percentage specific to *M. oryzae*.

### Novel and expanded findings in the CRZ1 regulatory circuitry

Our combined approach identified 140 direct targets of MoCRZ1 whose expression was concurrently regulated in a calcineurin/CRZ1 dependent manner. Sixty-two of these genes could be grouped in the same categories that were used to functionally assign yeast genes [32]. These groups include cell wall synthesis, ion or small molecule transport, vesicle transport, lipid or sterol synthesis, degradative enzymes, and signaling and transcription. This is expected as the *Δmocrz1* mutant also exhibited sensitivity to calcium ions and chemicals perturbing cell wall integrity as did the yeast *crz1* mutant [5]. However, the large diversity in the suite of individual target genes is compelling. In *S. cerevisiae*, genome wide expression profiling identified 153 calcium/calcineurin/Crz1p dependent genes [30]. Comparison between these two sets showed that only 15 out of 140 MoCRZ1 targets had 12 yeast orthologs whose expression was regulated by calcium/calcineurin/Crz1p (Table 3). When we compared our gene list to the 120 *A. fumigatus* genes whose expression was changed by exposure to calcium for 10 min [26], only 21 matched to 14 *A. fumigatus* genes, with only 6 having reciprocal best blast hits (Table 3). When the same analysis was applied to the 141 *A. fumigatus* genes whose expression was modulated by *AfcrzA*, as recently reported by Soriani et al. [43], 28 MoCRZ1 targets having 15 *A. fumigatus* orthologs were found with only 3 matching the 14 genes identified previously (data not shown). The observed diversity may reflect divergently evolved molecular features modulated by CRZ1 in each species to cope with its unique environment. This diversity was also reflected in the *crz1* mutant phenotypes across the species. For example, *crz1* mutants in different species showed a spectrum of ion sensitivity; *ΔcrzA* of A. *fumigatus* was more sensitive to Mn^2+^, but *Δmocrz1* of *M. oryzae* was not. *BcCRZ1* of *B. cinerea* was dispensable for conidia mediated infection, but *MoCRZ1* was necessary. These data suggest that although the core calcium signaling machinery including calmodulin and calcineurin is highly conserved across the species, their mechanism of action has diverged. Similar suggestions have been proposed by Karababa et al. [28]. Kraus and Heitman [44] also made note of species-specific action mechanisms through which calcium signaling involving calmodulin and calcineurin was manifested in three different species, *S. cerevisiae*, *C. albicans*, and *C. neoformans*. Additional evidence supporting this hypothesis is found in the differences in CDRE (calcineurin-dependent response elements) sequences [27], [30], [45], [46]. In *S. cerevisiae*, nucleotide stretches of 5′-GNGGC(G/T)CA-3′ was reported as the Crz1p-binding site by *in vitro* site selection. A similar sequence, 5′-GAGGCTG-3′, was also identified as a common motif in the upstream 500 bp regions of 40 genes with ≥4.0 fold Crz1-dependent expression profile [30]. Two similar sequences, 5′-GTGGCTC-3′ and 5′-GAGGCTC-3′, were reported as CDREs from the genus *Aspergillus* in the *A. nidulans* chsB and *A. giganteus* afp promoters [47]. A slightly divergent motif, 5′-G(C/T)GGT-3′, was identified as a common regulatory sequence from the 60 upstream regions of calcineurin/Crz1p-dependent genes of *C. albicans* [28]. In contrast, the motif sequences obtained in this study shows further divergence from that of *S. cerevisiae* or *C. albicans*. Although, core hepta nucleotide, 5′-GGCTC-3′, was found in the probe sequence of 10 genes including *PMC1* (MGG_02487) and MGG_03530 (chitin synthase activator), the occurrence of the full *S. cerevisiae* or *C. albicans* motif sequences is not enriched/overrepresented among the 106 sequences we analyzed (data not shown).

**Table 3:** List of common genes regulated by calcium/calcineurin/CRZ1 across species.

*M. oryzae*	Putative functions	*S. cerevisiae*	*A. fumigatus*
MGG_00289	amino-acid permease inda1		Afu4g09040
MGG_00504	zinc finger protein 740		Afu2g13770
MGG_00537	ammonium transporter MEP1	YGR121C	
MGG_01127	26S proteasome regulatory subunit-like protein		Afu1g13750 a
MGG_01150	calcineurin temperature suppressor Cts1		Afu2g13890
MGG_01494	conserved hypothetical protein	YMR016C	Afu4g10200
MGG_01802	chitin synthase 1	YNL192W	
**MGG_02487**	**PMC1, vacuolar Ca^2+^ transporting ATPase**	**YGL006W**	**Afu1g10880** b
MGG_02916	proteophosphoglycan ppg4	YMR016C	Afu4g10200
MGG_03218	calcineurin binding protein	YKL159c	Afu2g13060
**MGG_03307**	**hypothetical protein**	**YNL208W**	**Afu7g04870** b
MGG_03530	chitin synthase activator (Chs3)		Afu8g06700
MGG_03539	conserved hypothetical protein		Afu4g10200
MGG_03941	conserved hypothetical protein		Afu4g10200
MGG_04660	negative regulator of the PHO system	YOL016C	Afu1g05800
MGG_05133	MoCRZ1, C2H2 transcription factor		Afu2g13770
MGG_05723	fluconazole resistance protein 1	YOR273C	
MGG_06360	conserved hypothetical protein		Afu2g13890
MGG_06364	Zinc-finger transcription factor ace1 (ACEI)		Afu2g02080
MGG_06928	serine/threonine-protein kinase bur-1	YOL016C	Afu1g05800
MGG_07287	lysophospholipase 3	YOL011W	
MGG_07447	hypothetical protein	YGR189C	
MGG_09361	hypothetical protein, CgDN3	YNL208W	Afu1g11910
MGG_10058	predicted protein		Afu2g11840
MGG_10131	MSF superfamily multidrug-resistance protein	YOR273C	
MGG_10400	glucan 1,3-beta-glucosidase	YMR305C	
MGG_10869	MFS drug efflux transporter	YPR198W	
MGG_11178	Rho guanyl nucleotide exchange factor		Afu1g11910
MGG_12922	phospholipid-transporting ATPase 1	YGL006W	Afu3g10690

a.Blast best hit using 140 MoCRZ1 targets as a query was listed. Reciprocal best hit (RBH) was underlined.

b.Two genes sharing RBH among three species were emphasized as bold.

### Feedback regulation of the calcium signaling network by CRZ1

In addition to *PMC1*, several other genes implicated in the calcium signaling pathway regulating calcineurin activity were identified. Among them were calcineurin binding protein *CBP1* (MGG_03218) and the calcineurin temperature suppressor *CTS1* (MGG_01150), suggesting feedback regulation of calcineurin activity mediated by MoCRZ1. In addition, MoCRZ1 bound its own promoter to activate expression. *CBP1* shows homology to *CbpA* in *A. fumigatus* and *Cbp1* of *C. neoformans*, which in turn, are orthologous to *RCN1* of *S. cerevisiae*, an inhibitor of calcineurin called calcipressin. Over-expression of *RCN1* in a *pmc1* mutant background conferred Ca^2+^ tolerance by activation of vacuolar Ca^2+^/H^+^ exchanger *Vcx1p*, expression of which was negatively regulated by calcineurin. Expression of *RCN1* (YKL159c) was regulated by calcineurin and Crz1p, suggesting negative feedback regulation [30], [48]. However, both stimulatory and inhibitory regulation of calcineurin by *RCN1* was reported [49]. *RCN1* expression at endogenous level and phosphorylation by GSK-3 kinase, positively regulated calcineurin activity [49]. Degradation of phosphorylated *RCN1* is required for precise calcineurin activity in response to changes in Ca^2+^ concentration [50]. Negative feedback regulation of calcineurin by *CbpA* was also reported in *A. fumigatus*, where it down regulates *cnaA* expression as well as that of downstream genes *vcxA* and *chsA* encoding vacuolar Ca^2+^/H^+^ exchanger and chitin synthase A, respectively [51]. Expression of *CbpA* (Afu2g13060) was known to be up-regulated in response to Ca^2+^, which was CrzA dependent [26]. This feedback loop seems to extend at least to the level of calcineurin. Roles and action mechanisms of *Cbp1* in the calcium/calcineurin signaling pathway also seems to be diverged in a species specific manner. In *C. neoformans*, *Cbp1* does not stimulate or inhibit calcineurin expression, and does not seem to participate in a feedback loop. Taken together, these data lead us to propose that a negative or positive feedback loop, which includes MGG_03218, regulates the calcium/calcineurin signal transduction pathway in *M. oryzae*.

Phospholipid-binding protein Cts1 (calcineurin temperature suppressor) was identified and characterized in *C. neoformans* as able to restore growth defect at 37°C in calcineurin-deficient strains and to confer resistance to the calcineurin inhibitor FK506 [52]. Δ*cts*1 mutants were synthetically lethal in combination with a calcineurin mutation. However, no direct interaction between Cts1 with either the catalytic or regulatory subunit of calcineurin was reported. With these data, they suggested that Cts1 acted in either parallel pathways or a branched pathway to compensate, at least in part, for the loss of calcineurin function [52]. MGG_01150, an ortholog of *Cts1*, was found to be a direct target of MoCRZ1. Its calcineurin dependent expression pattern is opposite to that of *Cts1*. Unlike the elevated expression of *Cts1*in calcineurin deficient strains, MGG_01150 expression was activated by Ca^2+^ treatment, which was blocked by calcineurin inhibitor FK506 and abolished in the *Δmocrz1* mutant. Therefore, it is evident that MGG_01150 is a component of the calcineurin signaling pathway in *M. oryzae* unlike its counterpart in *C. neoformans*.

Our data revealed that MoCRZ1 binds to its own promoter to activate expression in a Ca^2+^ /calcineurin dependent manner. Therefore, MoCRZ1 regulation appears to occur at the posttranslational and transcriptional levels via activation by calcineurin and positive autoregulation respectively. Calcineurin/CRZ1 dependent expression of *CRZ1* was also reported in *C. albicans* suggesting a common mechanism of regulation across the fungal species [28]. However, expression dynamics of the catalytic (*MCNA*: MGG_07456) and regulatory (*MCNB*: MGG_06933) subunit were not altered in the *Δmocrz1* mutant compared to wild type in response to Ca^2+^ treatment (data not shown). This suggests that the proposed feedback regulation does not include direct regulation of calcineurin expression by CRZ1.

### Fungal pathogenicity

Involvement of CRZ1 in fungal virulence has been recently demonstrated in both human and plant pathogenic fungi [5], [10], [25], [26], [28], [29]. Signals related to these virulence traits seemed to be transmitted to a diverse range of downstream genes, as 18 genes out of 140 direct targets including MoCRZ1 have been found to be related to fungal virulence in both human and plant pathogens. Gene repertoire ranges from cell wall synthetic enzymes, proteins conferring resistance to antifungal agents encoded by major facilitator type transporter, calcium homeostasis to transcription factors. Three genes (*MoCRZ*1, MGG_03530 encoding chitin synthase activator 3, and MGG_02487) have been functionally characterized in *M. oryzae* [5], [8], [37]. Association of MGG_03530 encoding chitin synthase activator 3 (Chs3) with virulence was found in a T-DNA insertion strain with reduced growth rate on nutrient rich media, reduced conidiation rate with aberrant conidia morphology, and reduced appressorium formation and virulence [37]. Filamentous fungal genomes contain up to 10 chitin synthase genes of 7 classes [53]. Different CHS were regarded as to have functional redundancy in a variety of developmental processes because single mutation of a class I or II gene did not result in a marked phenotype. Therefore, specific roles for individual genes have not yet been clearly assigned and the association with fungal virulence has been controversial. However, several lines of evidence implicate class V myosin-like CHS in virulence in the maize anthracnose pathogen *Colletotrichum graminearum* [54] and in *Fusarium oxysporum*, the tomato wilt fungus [55]. One Class III chitinases, BcChs3a of *Botrytis cinerea* had important roles in virulence, especially on leaf tissue colonization, grape vines and *Arabidopsis thaliana* [56]. The *M. oryzae* genome contains 7 predicted chitin synthases, of which the expression of 5 were induced and 1 repressed in response to exogenous calcium treatment (data not shown). Only one (MGG_01802 encoding class II chitinase) was directly regulated by MoCRZ1. Therefore, calcium seems to regulate expression of most chitin synthases in diverse pathways.

Two genes encoding small molecule transporting P-type ATPases (MGG_02487 Ca transporting ATPase and MGG_12922 phospholipid-trasnporting ATPase) were found to have homologs implicated in fungal pathogenicity. Knock-down of MGG_02487 encoding PMC1 by RNAi technology resulted in no conidiation, growth retardation, and reduced melanization [8]. The association of *PMC1* and fungal virulence was not investigated in other fungi. Other evidence on the involvement of Ca^2+^ transporting ATPase in fungal virulence arises from the study of *PMR1* of *C. albicans* [57]. *PMR1* is a Golgi membrane located Ca^2+^ transporting P-type ATPase, and is known to work cooperatively with *PMC1* in the maintenance of cytosolic Ca^2+^ homeostasis. *Capmr1Δ* mutant of *C. albicans* had a weakened cell wall, probably due to the glycosylation defect and showed severely attenuated virulence in a murine model of systemic infection [57]. Two genes encoding Drs2 family of P-type ATPases, *PDE1* and *MgAPT2*, were functionally characterized to act in appressorium formation and invasive growth [34], [38]. *MgAPT2* was necessary for the normal development of Golgi apparatus that is required for secretion of a subset of extracellular enzymes via exocytosis [34], [58].

**Figure 6:**
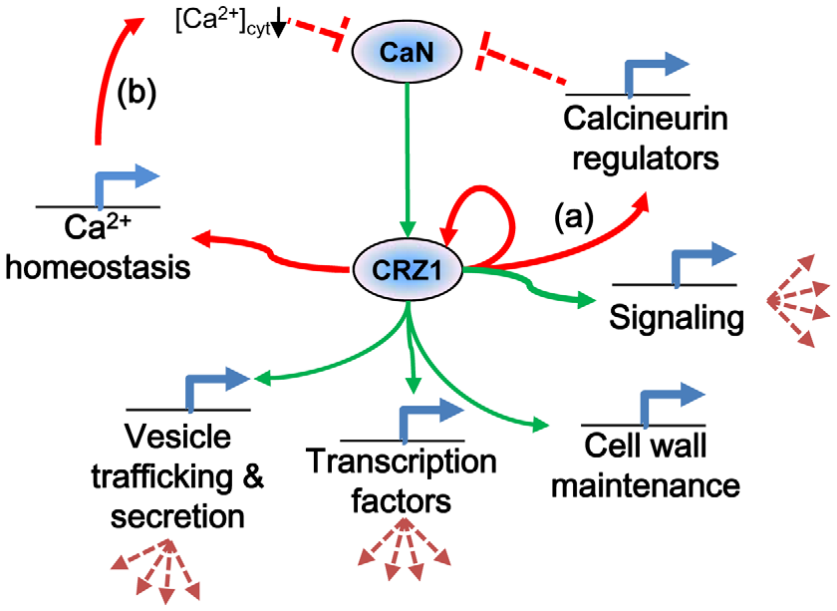
Proposed model of MoCRZ1 regulation of downstream genes. Consensus model of calcineurin/CRZ-1 signaling with green arrows indicating feedforward regulation and red arrows feedback regulation. Regulation of calcineurin occurs in multiple layers. (a) direct feedback by induction of expression of calcineurin regulators *CBP1* or *CTS1*. (b) indirect feedback by induction of *PMC1* leading to sequestration of cytosolic Ca^2+^ ions to inactivate calcineurin.

This study is the first of its kind where ChIP-chip technology has been applied to filamentous fungi. The correlation of comprehensive whole genome expression data with results from ChIP-chip have allowed for significant refinement of the predicted targets of MoCRZ1. This refinement alone allowed for the identification of a predicted signature binding motif for this transcription factor. This study reveals conserved elements of the calcium/calcineurin signaling pathway, as well as elucidates species specific differences that we propose function to regulate the system and allow for responses tailored to biology of the organism. Calcium signaling is a ubiquitous and complicated aspect of cell physiology. This study represents a major advance in our understanding of this pathway in *M. oryzae* and provides the launching point for the functional characterization of the genes and interactions it implicates. Figure 6 depicts our proposed model resulting from this work and includes our new findings of predictive roles for MoCRZ1 in autoregulation, feedback inhibition, and secretion.

## Materials and Methods

### Fungal cultures and chemical treatment

Strains of *M. oryzae* were maintained on oatmeal agar (50 g of oatmeal per liter) and grown at 22 °C under constant fluorescent light to promote conidiation. Mycelial blocks from actively growing margins of colony were inoculated into complete media (CM) liquid media at 25 °C by shaking for 3 days. After thorough washing with sterile distilled water, the mycelia were treated with 200 mM CaCl_2_ with or without 10 µg/ml FK506 for the indicated time. Mycelia were harvested and immediately frozen with liquid nitrogen and stored at −80°C before use. FK506 (Sigma, St. Louis, MO) stock solution in 5 mg/ml was prepared with DMSO, and stored at −20°C until used.

### Fungal transformation and microscopy

Protoplasts generation and transformation were performed following established protocols [59]. Protoplasts were generated from young mycelia grown in complete media with 10 mg/ml Lysing Enzyme (Sigma, St. Louis, MO) in 20% sucrose. Protoplasts were harvested by filtration through 4 layers of miracloth (Calbiochem, Darmstadt, Germany), washed twice with STC (20% sucrose, 50 mM Tris-HCl, 50 mM CaCl_2_, pH 8.0) followed by centrifugation at 5,000 rpm for 15 min at 4°C, and resuspended to 5×10^7^ protoplasts/ml. GFP tagging construct in TOPO cloning vector (P_MoCRZ1_::MoCRZ1::GFP) was co-transformed with pCX63 containing hygromycin resistance cassette by the mediation of 40% polyethyleneglycol. After incubation for 7–10 days at 25°C, hygromycin resistant colonies were transferred to V8 juice agar media. The fluorescing transformants observed under the microscope with epifluorescent optics (Nikon eclipse 80i, Melville, NY) were purified through single spore isolation. Nuclear translocalization of MoCRZ1 was observed after treatment with 200 mM CaCl_2_ with or without 10 µg/ml FK506 for 1 hour at room temperature.

### RNA isolation and real-time RT-PCR

Total RNA was isolated from frozen mycelial powder using an Easy-Spin RNA extraction kit (iNtRON Biotechnology, Seoul, Korea). Five micrograms of total RNA was reverse-transcribed into first-strand cDNA by oligo dT priming using the SuperScript first-strand cDNA synthesis kit according to the manufacturer's instructions (Invitrogen Life Technologies, Carlsbad, CA). Resulting cDNA was diluted to 1∶20 with sterile water. Real-time RT-PCR was performed according to the established protocol [59] using iQ SYBR Green Supermix (Bio-rad, Hercules, CA) on an iCycler iQ5 Real-Time PCR Detection System (Bio-rad). Fold changes were calculated by 2^−ΔΔCt^, where ΔΔCt  =  (Ct_gene_ _of interest_-Ct_control gene_)_test condition_-(Ct_gene_ _of interest_-Ct_control gene_)_control condition_. Test and control conditions are as same as in Figure 3A, where (a) compares expression level between Ca^2+^ treated vs. no treatment in wild type strain KJ201, (b) Ca^2+^ vs. Ca^2+^+FK506 in KJ201, (c) Ca^2+^ treatments in KJ201 vs. in *Δmocrz1*. Primer sequences were listed in Table S5.

### Chromatin immunoprecipitation-chip and analysis

Young mycelia grown in liquid media were treated with 200 mM CaCl_2_ with or without 10 µg/ml FK506 for 1 hour with shaking. The harvested mycelia were divided with one part being immediately frozen in liquid nitrogen for future RNA isolation and the other treated with 1% formaldehyde in buffer A (0.4 M sucrose, 10 mM Tris-HCl, pH 8.0, 1 mM EDTA, 1 mM phenylmethylsulfonyl fluoride, and 1% formaldehyde) for cross-linking for 20 min. Mycelia were harvested with excess amount of distilled water after stopping cross-link with 0.1 M glycine for 10 min, frozen in liquid nitrogen, ground into a fine powder in a chilled mortar and pestle, and stored at −80°C until used. Chromatin immunoprecipitation was conducted according to published procedures with modification [60], [61]. Nuclear DNA was then isolated from cross-linked mycelia with Plant Nuclear Isolation Kit (Sigma, St. Louis, MO) and sheared into fragments by sonication to 200- to 1,000-bp with an average size of 500 bp. Sonication was conducted on ice with an amplitude of 10% using 30×30 s pulses (30 s between bursts) using Biorupter (Cosmo Bio, Tokyo, Japan). After pre-clearing nuclear lysates with Salmon sperm/protein A agarose (Upstate, Temecula, CA) for 4 hours at 4°C, immunoprecipitations were performed with either 1 µg of rabbit control IgG (ab46540-1, Abcam, Cambridge, MA) or 0.5 µl of antiGFP antibody (ab290, Abcam) for overnight at 4°C. A small aliquot of DNA (30%) was saved for input DNA (input). Immunoprecipitated DNA was captured with proteinA agarose beads (Upstate, Temecula, CA) for 4 hours, and then washed twice with LNDET buffer (0.25 M LiCl, 1% NP40, 1% deoxycholate, 1 mM EDTA) and twice with TE buffer. The DNAs were reverse-cross linked at 65 °C overnight in elution buffer (1% SDS and 0.1 M NaHCO_3_) containing 1 mg/ml proteinase K, and purified using PCR purification kit (Qiagen). Real-time PCR was performed with 1 µl each of pulled-down DNA and input DNA as template following the procedures described above. Fold changes for control gene (*β-tubulin*) and putative target gene (*PMC1*) were calculated by 2^−ΔΔCt^, where ΔΔCt  = (Ct_input DNA_-Ct_ChIPed DNA_) _Ca2+ treated sample_ - (Ct_input DNA_-Ct_ChIPed DNA_) _Ca2+/FK506 treated sample_. Primer sequences for the promoter region of *PMC1* and *β-tubulin* were listed in Table S5. For ChIP-chip experiments, 10 µl ChIPed DNA and 10 ng input DNA were amplified using GenomePlex Whole Genome Amplification Kit (Sigma). Amplified DNA was then labeled with Cy3 or Cy5 fluorescent dyes for input or immunoprecipitated DNA, respectively, and hybridized to NimbleGen *Magnaporthe grisea* promoter tiling arrays according to the manufacturer's instruction (NimbleGen Systems of Iceland). Probes for tiling array were designed to have about 70 nucleotides per 100 bp of promoter and intergenic region based on annotation of *M. grisea* genome version 5. After getting peak intensity, peak data files (.gff) were generated from the scaled log2-ratio data using NimbleScan. It detects peaks by searching for 4 or more probes whose signals are above the cutoff values using a 500 bp sliding window. The ratio data was then randomized 20 times to evaluate the probability of “false positives”. Each peak was then assigned a false discovery rate (FDR) score based on the randomization. Peaks with FDR score ≤0.2 were regarded as positive.

### Expression microarray and analysis

Mycelia of wild type KJ201 and *Δmocrz1* strain were treated with 200 mM CaCl_2_ with or without 10 µg/ml FK506, with water as control. Initially, samples were harvested at 0, 15, 30, and 60 min. after treatment. *PMC1* expression level was checked by RT-PCR with the highest expression at the 30 min. time point. Four biological replicates of wild type and mutant mycelia were harvested after 30 min. treatment with chemicals, frozen immediately with liquid nitrogen. Total RNA was isolated described as above. After validation of sample quality by RT-PCR, total RNA was sent to Cogenics (Morrisville, NC) for hybridization to the Agilent *M. grisea* whole genome microarray chip version 2.0 using the single channel hybridization design. Quality of RNA was determined with Agilent Bioanalyzer. Five hundred nanograms of total RNA was converted into labeled cRNA with nucleotides coupled to fluorescent dye Cy3 using the Quick Amp Kit following the manufacturer's instructions (Agilent Technologies, Palo Alto, CA). After analyzing the quality with Agilent Bioanalyzer, Cy3-labeled cRNA (1.65 µg) was hybridized to *M. grisea* 2.0 4×44 k microarrays. The hybridized array was washed and scanned, and the data were extracted from the scanned image using Feature Extraction version 10.2 (Agilent Technologies). An error-weighted average signal intensity of two probes within a chip was used for normalization with Lowess normalization module implanted in JMP Genomics software. An average expression of all probes among 16 data sets was used as the baseline. Pairwise comparison between treatments was conducted to get the expression profiles of each probe. Genes were regarded as differentially expressed if their average signal intensity among 4 replicates was above 20 in a minimum of one condition and expression ratio is greater than 2 fold with P<0.05 (Student's t-test).

### Motif analysis

The two commonly used motif discovery programs, MEME [39] and MDScan [40], were used to identify the MoCRZ1 binding motif. Input data consisted of the exact binding sequences retrieved from the promoters of 83 genes with differential expression in the WT/*Δmocrz1* comparison (Figure 3A). Candidate motifs from both algorithms were manually interrogated and enumerated to identify the 3 top candidates, and queried against the yeast motif database using TOMTOM [41]. Enrichment was calculated over the 106 background sequence set which was randomly retrieved from intergenic region of the whole genome. Consensus motif sequences were calculated using WebLogo server at http://weblogo.berkeley.edu [62].

### Protein expression and electrophoretic mobility shift assay

Protein expression vector was constructed by ligation of *MoCRZ1* cDNA encompassing full ORF into pGEX-6P-1 (Invitrogen, Carlsbad, CA) having GST tag at the N terminus. The resulting construct was transformed into the *E. coli* strain BL21 (DE3) pLysS (Novagen) after verifying the cDNA sequences. Induced protein was purified with GST agarose beads (Sigma) based on the procedures of Frangioni et al.[63].

Probe DNA was prepared by PCR with Biotin labeled primer at the 5′ end, followed by gel purification. Cold probe was amplified with the same primer sequence without Biotin labeling. Primer sequences were listed in Table S5. Binding of putative motif sequences to MoCRZ1 protein was performed using LightShift Chemiluminescent EMSA kit following the manufacturer's manual (PIERCE, Rockford, IL). Reaction mixtures containing 10 ng of purified MoCRZ1 and biotin labeled probe were incubated for 20 min. at room temperature. The reactions were electrophoresed on 5% polyacrylamide gel in 0.5×TBE, and transferred to a positively charged nylon membrane (Hybond N+, GE Healthcare). Signals were detected using Chemiluminescent Nucleic Acid Detection Module (PIERCE) according to the manufacturer's instruction.

## Supporting Information

Table S1 List of genes identified from ChIP-chip analysis.
(0.03 MB PDF)

Table S2 Number of genes with GO terms in each group.
(0.04 MB XLS)

Table S3 GO annotation of MoCRZ1 direct targets.
(0.05 MB XLS)

Table S4 Genes with putative MoCRZ1 binding motifs in the promoter region.
(0.03 MB XLS)

Table S5 Primers used in this study.
(0.04 MB XLS)
