# Supplementary material for: Combining ChIP-chip and Expression Profiling to Model the MoCRZ1 Mediated Circuit for Ca2+/Calcineurin Signaling in the Rice Blast Fungus
Source: PLoS Pathog. 2010 May 20;6(5):e1000909. doi: 10.1371/journal.ppat.1000909 (PMC2873923; doi:10.1371/journal.ppat.1000909)
Supplement: Table S1 — List of genes identified from ChIP-chip analysis. (0.03 MB PDF) [file ppat.1000909.s001.pdf]

Table S1. List of genes identified from ChIP-chip analysis											
MG5	Putative function	CA_Ave	CAFK_Av	CK_Ave.	CRZ_Ave	CA/CK	TT(CA/CK	CA/CAFK	T(CA/CAF	CA/CRZ	TT(CA/CRZ)
MGG_00052	hypothetical protein	122256	73590	73032	59215	1.7	0.034	1.7	0.025	2.1	0.005
MGG_00067	glycerol-3-phosphate dehydrogenase	18665	16922	7949	20894	2.3	0.021	1.1	0.600	0.9	0.554
MGG_00133	siderophore iron transporter mirC	2380	3185	4150	1817	0.6	0.020	0.7	0.170	1.3	0.086
MGG_00160	predicted protein	11	17	15	12	0.7	0.276	0.6	0.063	0.9	0.817
MGG_00197	predicted protein	338	364	377	577	0.9	0.814	0.9	0.829	0.6	0.018
MGG_00198	bacterial hemoglobin	111070	167407	164203	129672	0.7	0.184	0.7	0.057	0.9	0.170
MGG_00288	G-patch domain protein (TFIP11), putative	1742	576	698	812	2.5	0.005	3.0	0.003	2.1	0.007
MGG_00289	amino-acid permease inda1	1927	4146	2829	2073	0.7	0.372	0.5	0.049	0.9	0.745
MGG_00297	conserved hypothetical protein	10773	10726	2832	9102	3.8	0.013	1.0	0.992	1.2	0.461
MGG_00298	conserved hypothetical protein	90405	20864	12820	18268	7.1	0.052	4.3	0.014	4.9	0.010
MGG_00310	predicted protein	123	58	60	9	2.1	0.324	2.1	0.327	13.7	0.140
MGG_00352	conserved hypothetical protein	254	181	182	76	1.4	0.325	1.4	0.096	3.3	0.001
MGG_00433	predicted protein	1537	1991	2718	1135	0.6	0.012	0.8	0.068	1.4	0.144
MGG_00450	phosphoenolpyruvate carboxykinase	6137	9317	12480	3861	0.5	0.301	0.7	0.124	1.6	0.112
MGG_00504	zinc finger protein 740	3206	3446	1189	2720	2.7	0.010	0.9	0.568	1.2	0.390
MGG_00537	ammonium transporter MEP1	10350	8936	21823	8158	0.5	0.000	1.2	0.492	1.3	0.179
MGG_00538	acid phosphatase	840	731	929	542	0.9	0.527	1.1	0.359	1.5	0.021
MGG_00551	laccase-3	1863	623	1620	368	1.1	0.757	3.0	0.000	5.1	0.000
MGG_00552	acid phosphatase	6042	4085	4470	1816	1.4	0.421	1.5	0.046	3.3	0.001
MGG_00592	cell wall glucanosyltransferase Mwg1	36583	46658	29657	41135	1.2	0.137	0.8	0.028	0.9	0.303
MGG_00595	myc-type bHLH transcription factor Cph2	1743	2527	3140	2046	0.6	0.017	0.7	0.019	0.9	0.234
MGG_00602	cross-pathway control protein 1	219	336	224	275	1.0	0.904	0.7	0.014	0.8	0.004
MGG_00603	predicted protein	45	55	36	64	0.8	0.569	1.3	0.534	0.7	0.192
MGG_00767	cysteine desulfurase	15609	13593	6631	9635	2.4	0.023	1.1	0.521	1.6	0.079
MGG_00776	DUF689 domain protein	6702	9104	6163	8551	1.1	0.724	0.7	0.283	0.8	0.458
MGG_00909	conserved hypothetical protein	141	162	128	153	1.1	0.468	0.9	0.420	0.9	0.498
MGG_00910	predicted protein	4	4	4	4	1.1	0.132	1.1	0.087	1.1	0.140
MGG_01045	arrestin domain-containing protein	535	671	706	616	0.8	0.155	0.8	0.189	0.9	0.484
MGG_01057	PtaB protein, putative	1315	1443	834	965	1.6	0.142	0.9	0.447	1.4	0.025
MGG_01061	coproporphyrinogen III oxidase	701	1005	1392	857	0.5	0.052	0.7	0.225	0.8	0.330
MGG_01127	26S proteasome regulatory subunit-like protein	7622	20047	11626	5871	0.7	0.250	0.4	0.010	1.3	0.237
MGG_01141	estradiol 17-beta-dehydrogenase 12	137	74	123	118	1.1	0.808	1.9	0.213	1.2	0.697
MGG_01150	calcineurin temperature suppressor Cts1	17820	3302	3208	2226	5.6	0.010	5.4	0.012	8.0	0.007
MGG_01151	integral membrane protein	412	304	301	368	1.4	0.316	1.4	0.400	1.1	0.660
MGG_01159	histone H3	117	131	138	67	0.8	0.524	0.9	0.772	1.7	0.050
MGG_01243	predicted protein	1293	812	765	433	1.7	0.001	1.6	0.013	3.0	0.000
MGG_01461	conserved hypothetical protein	31680	53535	41015	57707	0.8	0.196	0.6	0.031	0.5	0.005
MGG_01489	conserved hypothetical protein	4129	3740	8611	3114	0.5	0.003	1.1	0.525	1.3	0.113
MGG_01490	conserved hypothetical protein	6225	7098	21681	5459	0.3	0.024	0.9	0.816	1.1	0.714

MGG_01494	conserved hypothetical protein	712	326	156	232	4.6	0.022	2.2	0.045	3.1	0.015
MGG_01585	translocator protein	27	83	27	25	1.0	0.978	0.3	0.003	1.1	0.752
MGG_01586	arsenite resistance protein Ars2, putative	11720	10837	5880	10432	2.0	0.011	1.1	0.463	1.1	0.428
MGG_01729	conserved hypothetical protein	16208	14662	23422	15079	0.7	0.031	1.1	0.732	1.1	0.616
MGG_01777	C6 zinc finger domain-containing protein	742	666	610	487	1.2	0.358	1.1	0.345	1.5	0.006
MGG_01778	aflatoxin efflux pump	3685	2753	3822	2158	1.0	0.837	1.3	0.249	1.7	0.054
MGG_01790	acyl-CoA desaturase	36099	43409	45265	29418	0.8	0.470	0.8	0.046	1.2	0.036
MGG_01802	chitin synthase 1	12513	6908	3018	3452	4.1	0.003	1.8	0.040	3.6	0.002
MGG_01825	conserved hypothetical protein	5240	7559	2297	7712	2.3	0.110	0.7	0.473	0.7	0.198
MGG_01826	cell cycle control protein (Cwf26), putative	7273	7099	7650	8870	1.0	0.719	1.0	0.796	0.8	0.094
MGG_01872	hypothetical protein	13457	5163	3357	6568	4.0	0.012	2.6	0.011	2.0	0.022
MGG_01925	bifunctional P-450:NADPH-P450 reductase	341648	389042	364540	401292	0.9	0.714	0.9	0.256	0.9	0.082
MGG_01944	hypothetical protein	43380	17763	22655	28572	1.9	0.168	2.4	0.092	1.5	0.235
MGG_02401	HIT finger domain protein, putative	18267	13632	4760	18185	3.8	0.007	1.3	0.247	1.0	0.979
MGG_02402	hypothetical protein	104029	8362	10818	14133	9.6	0.042	12.4	0.004	7.4	0.006
MGG_02450	conserved hypothetical protein	1210	1200	2143	986	0.6	0.093	1.0	0.979	1.2	0.249
MGG_02474	C2H2 transcription factor, putative	939	770	397	920	2.4	0.060	1.2	0.676	1.0	0.922
MGG_02487	PMC1	9254	1850	1034	538	8.9	0.002	5.0	0.001	17.2	0.000
MGG_02630	pH-response regulator protein palI/RIM9	661	579	413	470	1.6	0.000	1.1	0.306	1.4	0.013
MGG_02806	ATP-dependent RNA helicase SUB2	11321	7012	6762	6263	1.7	0.001	1.6	0.001	1.8	0.000
MGG_02807	RNAse P Rpr2/Rpp21 subunit domain-containing pr	770	333	193	253	4.0	0.006	2.3	0.017	3.0	0.004
MGG_02858	predicted protein	25	22	32	29	0.8	0.090	1.1	0.190	0.9	0.291
MGG_02905	tRNA-specific adenosine deaminase 2	460	576	344	413	1.3	0.198	0.8	0.296	1.1	0.524
MGG_02906	conserved hypothetical protein	30345	26438	17949	23828	1.7	0.014	1.1	0.224	1.3	0.129
MGG_02916	predicted protein	20855	16245	5373	17850	3.9	0.004	1.3	0.165	1.2	0.455
MGG_02917	hypothetical protein	44308	25612	11676	20242	3.8	0.009	1.7	0.085	2.2	0.028
MGG_03054	TAM domain methyltransferase, putative	80	63	141	74	0.6	0.077	1.3	0.313	1.1	0.771
MGG_03057	WD repeat-containing protein	1213	607	1035	460	1.2	0.525	2.0	0.088	2.6	0.015
MGG_03132	DNA repair helicase RAD3	239	372	250	385	1.0	0.802	0.6	0.183	0.6	0.050
MGG_03133	hypothetical protein (epithelial zinc-finger	21228	21792	9019	31812	2.4	0.060	1.0	0.921	0.7	0.105
MGG_03200	zinc metalloprotease mde10 precursor	4885	3508	4098	1675	1.4	0.057	1.2	0.449	2.9	0.000
MGG_03218	calcineurin binding protein, putative	153058	11350	17231	5605	13.5	0.002	8.9	0.001	27.3	0.002
MGG_03288	bZIP transcription factor, putative	138648	138874	61851	76903	2.2	0.002	1.0	0.993	1.8	0.006
MGG_03289	predicted protein	56	37	40	37	1.5	0.196	1.4	0.319	1.5	0.166
MGG_03291	related to SDA1 protein	1316	1340	513	1015	2.6	0.028	1.0	0.950	1.3	0.332
MGG_03292	conserved hypothetical protein	53686	35328	10944	49477	4.9	0.002	1.5	0.073	1.1	0.668
MGG_03307	glutamine-serine-proline rich protein, putative	300545	147968	112984	95997	2.7	0.002	2.0	0.004	3.1	0.001
MGG_03325	predicted protein	362	145	96	89	3.8	0.016	2.5	0.032	4.1	0.006
MGG_03330	dimethylaniline monooxygenase	3208	2658	7638	2924	0.4	0.005	1.2	0.510	1.1	0.659
MGG_03333	hypothetical protein	32669	33211	42103	17995	0.8	0.411	1.0	0.952	1.8	0.030
MGG_03470	hypothetical protein	35660	3081	8611	1019	4.1	0.016	11.6	0.007	35.0	0.006

MGG_03471	predicted protein	33	9	14	7	2.4	0.010	3.7	0.003	5.0	0.002
MGG_03530	chitin synthase activator (Chs3)	15421	5011	2654	3088	5.8	0.012	3.1	0.011	5.0	0.004
MGG_03539	conserved hypothetical protein	4856	2350	6398	1300	0.8	0.389	2.1	0.054	3.7	0.005
MGG_03615	cytoplasmic protein required for cell viability	415	420	259	732	1.6	0.092	1.0	0.971	0.6	0.008
MGG_03668	importin subunit beta-1	1233	2056	577	1597	2.1	0.006	0.6	0.229	0.8	0.096
MGG_03674	predicted protein	762	623	867	439	0.9	0.619	1.2	0.115	1.7	0.002
MGG_03675	conserved hypothetical protein	2826	5988	3575	1760	0.8	0.621	0.5	0.007	1.6	0.103
MGG_03678	AT hook motif protein	616	503	814	520	0.8	0.201	1.2	0.184	1.2	0.132
MGG_03691	conserved hypothetical protein	74853	65027	71614	103883	1.2	0.499	1.0	0.805	0.7	0.074
MGG_03692	glutaminyl-peptide cyclotransferase	2518	1346	1632	2916	1.5	0.165	1.9	0.002	0.9	0.186
MGG_03703	cell polarity protein	5061	4027	2227	3481	2.3	0.056	1.3	0.421	1.5	0.151
MGG_03837	telomere silencing protein Zds1	6351	8647	3679	5516	1.7	0.103	0.7	0.301	1.2	0.422
MGG_03863	hypothetical protein	98	42	57	68	1.7	0.051	2.3	0.000	1.4	0.025
MGG_03864	hypothetical protein	2501	2174	2081	3363	1.2	0.475	1.2	0.637	0.7	0.116
MGG_03887	conserved hypothetical protein	815	1120	1323	774	0.6	0.084	0.7	0.127	1.1	0.764
MGG_03913	predicted protein	42	41	28	77	1.5	0.247	1.0	0.882	0.5	0.000
MGG_03914	hypothetical protein	39469	35811	7890	27348	5.0	0.005	1.1	0.708	1.4	0.078
MGG_03920	inositolphosphorylceramide-B C-26 hydroxylase	9782	9649	15480	8599	1.0	0.940	0.6	0.009	1.1	0.268
MGG_03937	serine/threonine protein kinase	2493	2526	990	810	2.5	0.018	1.0	0.929	3.1	0.001
MGG_03939	pathway-specific nitrogen regulator	1016	1172	1649	1145	0.6	0.093	0.9	0.580	0.9	0.344
MGG_03940	aminotransferase, classes I and II,	3294	2544	3569	4774	0.9	0.629	1.3	0.439	0.7	0.196
MGG_03941	conserved hypothetical protein	3196	79	320	156	10.0	0.122	40.3	0.043	20.5	0.021
MGG_04085	AAA family ATPase	401015	297145	160900	285543	2.5	0.001	1.3	0.241	1.4	0.012
MGG_04090	SNARE protein [Neurospora crassa OR74A])	6899	4551	3825	2909	1.8	0.016	1.5	0.133	2.4	0.000
MGG_04104	60S ribosomal protein L22	5273	5326	6838	10139	0.8	0.183	1.0	0.966	0.5	0.107
MGG_04105	conserved hypothetical protein	54989	53974	15794	67012	1.0	0.932	3.5	0.006	0.8	0.360
MGG_04156	aspartate aminotransferase	17789	30332	27553	23759	0.6	0.007	0.6	0.001	0.7	0.035
MGG_04215	homocysteine S-methyltransferase	953	403	364	429	2.6	0.003	2.4	0.004	2.2	0.004
MGG_04237	conserved hypothetical protein	18261	10347	17292	24238	1.1	0.684	1.8	0.042	0.8	0.027
MGG_04242	bax Inhibitor family protein	12815	18249	11176	10023	1.1	0.737	0.7	0.277	1.3	0.229
MGG_04362	hypothetical protein	12428	6379	3968	4662	1.9	0.005	3.1	0.004	2.7	0.001
MGG_04428	zinc finger transcription factor ace1	62927	115752	41085	56482	1.5	0.088	0.5	0.035	1.1	0.439
MGG_04643	chalcone synthase C	552	708	458	577	1.2	0.402	0.8	0.035	1.0	0.755
MGG_04660	negative regulator of the PHO system	22484	12221	7904	4930	2.8	0.001	1.8	0.027	4.6	0.000
MGG_04683	15-hydroxyprostaglandin dehydrogenase	13508	17423	12301	18817	1.1	0.748	0.8	0.342	0.7	0.059
MGG_04764	cystathionine gamma-synthase	14941	17842	15507	16747	1.0	0.805	0.8	0.161	0.9	0.619
MGG_04768	conserved hypothetical protein	9158	6504	12400	4022	0.7	0.206	1.4	0.157	2.3	0.003
MGG_04804	hypothetical protein	1864	1598	1592	885	1.2	0.650	1.2	0.450	2.1	0.010
MGG_04933	hypothetical protein	1958	2805	1337	2741	1.5	0.117	0.7	0.072	0.7	0.088
MGG_04975	alcohol dehydrogenase	32	77	29	48	1.1	0.755	0.4	0.200	0.7	0.114
MGG_05084	conserved hypothetical protein	16	19	27	10	0.6	0.118	0.8	0.602	1.6	0.280

MGG_05085	MFS transporter, putative	1330	1049	2330	964	0.6	0.003	1.3	0.334	1.4	0.141
MGG_05128	dicarboxylic amino acid permease	3073	5235	2490	4402	1.2	0.113	0.6	0.004	0.7	0.038
MGG_05133	C2H2 type zinc finger domain-containing protein	52398	29516	9518	1670	5.5	0.006	1.8	0.103	31.4	0.029
MGG_05134	eukaryotic translation initiation factor 3	3494	6007	2751	7577	1.3	0.192	0.6	0.010	0.5	0.002
MGG_05169	tRNA ligase	23165	23087	11134	21908	2.1	0.032	1.0	0.991	1.1	0.776
MGG_05183	cript family protein	2085	881	975	1022	2.1	0.025	2.4	0.007	2.0	0.012
MGG_05344	probable SnodProt1 PRECURSOR	123138	159690	162788	181392	0.8	0.265	0.8	0.171	0.7	0.053
MGG_05356	AF485329_1 AcrB	1406	1703	1066	1069	1.3	0.023	0.8	0.165	1.3	0.051
MGG_05368	Ankyrin repeat protein	1298	1559	2342	1703	0.6	0.187	0.8	0.592	0.8	0.167
MGG_05402	similar to collagen, type XV, alpha 1	103	57	49	76	2.1	0.018	1.8	0.093	1.4	0.267
MGG_05525	UPF0187 domain membrane protein	12502	6719	5816	3745	2.1	0.005	1.9	0.007	3.3	0.000
MGG_05537	AAA ATPase central domain-containing protein	13835	16197	13701	15347	1.0	0.934	0.9	0.476	0.9	0.624
MGG_05621	T50987 related to prefoldin subunit	4	9	4	11	1.1	0.669	0.5	0.106	0.4	0.024
MGG_05723	fluconazole resistance protein 1	4659	18297	8624	6095	0.5	0.056	0.3	0.011	0.8	0.435
MGG_05727	ankyrin repeat protein	1413	1608	492	1423	2.9	0.015	0.9	0.514	1.0	0.970
MGG_05741	isochorismatase domain-containing protein 2	566	616	1118	830	0.5	0.030	0.9	0.666	0.7	0.069
MGG_05806	hypothetical protein	1159	455	1821	404	0.6	0.177	2.5	0.019	2.9	0.007
MGG_05807	conserved hypothetical protein	1117	1143	852	1693	1.3	0.312	1.0	0.922	0.7	0.076
MGG_05925	predicted protein	133	560	77	197	1.7	0.347	0.2	0.344	0.7	0.312
MGG_06090	conserved hypothetical protein	6495	1912	1150	1625	5.6	0.010	3.4	0.006	4.0	0.005
MGG_06185	ribosomal protein P1	2034	1854	3059	4535	1.1	0.772	0.7	0.257	0.4	0.108
MGG_06190	DNA-3-methyladenine glycosylase	9	8	19	10	0.5	0.117	1.1	0.809	0.8	0.742
MGG_06237	SET domain protein	1039	288	327	514	3.2	0.004	3.6	0.001	2.0	0.006
MGG_06238	conserved hypothetical protein	29510	10280	10445	14135	2.8	0.002	2.9	0.002	2.1	0.005
MGG_06301	conserved hypothetical protein	2396	5167	1390	2996	0.5	0.027	1.7	0.020	0.8	0.090
MGG_06311	DNA-directed RNA polymerase I and III 14 KDA poly	4042	6290	7322	6666	0.6	0.007	0.6	0.038	0.6	0.027
MGG_06312	C6 zinc finger domain-containing protein	1316	4863	1284	3082	1.0	0.961	0.3	0.001	0.4	0.010
MGG_06359	conserved hypothetical protein	23882	7666	3228	2360	7.4	0.017	3.1	0.022	10.1	0.005
MGG_06360	conserved hypothetical protein	1091	379	519	303	2.1	0.018	2.9	0.004	3.6	0.002
MGG_06364	ACE1_TRIRE Zinc-finger transcription factor ace1 (/	1206	495	238	1015	2.4	0.014	5.1	0.024	1.2	0.428
MGG_06411	conserved hypothetical protein	954	889	906	2137	1.1	0.883	1.1	0.828	0.4	0.005
MGG_06412	conserved hypothetical protein	98297	90630	77989	138447	1.3	0.220	1.1	0.559	0.7	0.018
MGG_06469	potassium transport protein 1	3639	3131	3659	1530	1.0	0.952	1.2	0.339	2.4	0.000
MGG_06473	conserved hypothetical protein	11717	10448	13514	11192	0.9	0.081	1.1	0.051	1.0	0.823
MGG_06484	hypothetical protein	13837	42361	15174	11785	0.9	0.806	0.3	0.047	1.2	0.589
MGG_06538	Bys1 family protein	289315	321343	395769	325084	0.7	0.200	0.9	0.634	0.9	0.533
MGG_06638	hypothetical protein	5514	5519	7086	6150	0.8	0.147	1.0	0.997	0.9	0.481
MGG_06928	serine/threonine-protein kinase bur-1	818	541	146	633	5.6	0.002	1.5	0.052	1.3	0.218
MGG_07044	hypothetical protein	2351	3994	4077	4176	0.6	0.160	0.6	0.126	0.6	0.026
MGG_07075	ATPase family AAA domain-containing protein 1-A	3055	2596	1050	2219	2.9	0.010	1.2	0.582	1.4	0.255
MGG_07076	dehydrogenase/reductase SDR family member	1316	1341	814	1957	1.6	0.041	1.0	0.956	0.7	0.051

MGG_07077	golgi apyrase	7565	3588	5008	5395	1.5	0.041	2.1	0.008	1.4	0.061
MGG_07100	predicted protein	5281	1238	1685	279	3.1	0.018	4.3	0.035	18.9	0.004
MGG_07102	conserved hypothetical protein	8931	16388	8057	17527	1.1	0.672	0.5	0.035	0.5	0.016
MGG_07144	hypothetical protein	39822	31186	9370	46103	4.3	0.006	1.3	0.546	0.9	0.466
MGG_07230	alpha-1,3-mannosyltransferase CMT1	988	41	94	57	10.5	0.026	24.1	0.022	17.4	0.002
MGG_07231	conserved hypothetical protein	42902	4826	24618	2247	1.7	0.166	8.9	0.012	19.1	0.031
MGG_07234	FK506-binding protein 2	8571	8405	10803	9451	0.8	0.357	1.0	0.929	0.9	0.581
MGG_07236	predicted protein	39	35	36	33	1.1	0.814	1.1	0.520	1.2	0.198
MGG_07237	conserved hypothetical protein	45100	44687	28618	28430	1.0	0.960	1.6	0.015	1.6	0.010
MGG_07287	lysophospholipase 3	388	354	79	118	4.9	0.005	1.1	0.751	3.3	0.002
MGG_07314	C2H2 finger domain protein	4197	6028	2786	4865	1.5	0.120	0.7	0.061	0.9	0.350
MGG_07340	epoxide hydrolase	191	103	70	43	2.7	0.245	1.9	0.397	4.4	0.160
MGG_07387	conserved hypothetical protein	245	181	201	187	1.2	0.344	1.4	0.035	1.3	0.046
MGG_07447	hypothetical protein	1330	905	616	652	2.2	0.003	1.5	0.031	2.0	0.001
MGG_07479	Kelch motif family protein	14017	10492	9136	7907	1.5	0.108	1.3	0.229	1.8	0.051
MGG_07482	FK506 suppressor Sfk1	903	1158	584	528	1.5	0.088	0.8	0.470	1.7	0.112
MGG_07518	centromere/microtubule-binding protein cbf5	2248	2581	2564	1641	0.9	0.636	0.9	0.649	1.4	0.294
MGG_07535	rhomboid family membrane protein	1813	483	505	438	3.6	0.005	3.8	0.001	4.1	0.001
MGG_07603	conserved hypothetical protein	8717	3643	2446	1051	3.6	0.038	2.4	0.061	8.3	0.005
MGG_07604	conserved hypothetical protein	20738	16579	28058	24051	0.7	0.068	1.3	0.240	0.9	0.125
MGG_07606	general amino-acid permease GAP1	17816	22654	32576	16043	0.5	0.016	0.8	0.091	1.1	0.464
MGG_07654	prolyl peptidase	21482	18583	22054	12590	1.0	0.910	1.2	0.678	1.7	0.012
MGG_07684	predicted protein	43	35	76	29	0.6	0.030	1.2	0.255	1.5	0.076
MGG_07747	UPF0145 domain protein	7808	2828	2827	5999	2.8	0.175	2.8	0.055	1.3	0.433
MGG_07765	conserved hypothetical protein	1461	1101	633	836	2.3	0.031	1.3	0.309	1.7	0.061
MGG_07791	surface protein 1	370774	287472	390184	388143	1.3	0.195	1.0	0.756	1.0	0.773
MGG_07792	predicted protein	454	369	466	770	1.2	0.544	1.0	0.901	0.6	0.049
MGG_08003	UbiA prenyltransferase	3689	1681	573	994	6.4	0.253	2.2	0.322	3.7	0.136
MGG_08019	F-box domain-containing protein	121473	100791	209955	62830	1.2	0.455	0.6	0.006	1.9	0.015
MGG_08024	hypothetical protein	2290	565	614	296	3.7	0.005	4.1	0.001	7.7	0.001
MGG_08045	conserved hypothetical protein	1818	1996	3347	1729	0.5	0.218	0.9	0.879	1.1	0.808
MGG_08046	bilirubin oxidase	1315	1949	1143	801	1.2	0.718	0.7	0.531	1.6	0.221
MGG_08055	conserved hypothetical protein	17321	27039	27518	15800	0.6	0.276	0.6	0.317	1.1	0.709
MGG_08056	uric acid-xanthine permease	102	107	119	85	0.9	0.482	1.0	0.850	1.2	0.416
MGG_08072	cholesterol oxidase	3101	4820	979	1857	3.2	0.004	0.6	0.037	1.7	0.025
MGG_08112	Patatin-like serine hydrolase	4458	4874	2749	4510	1.6	0.006	0.9	0.057	1.0	0.938
MGG_08113	hypothetical protein	23058	25407	22176	42876	1.0	0.872	0.9	0.802	0.5	0.026
MGG_08202	conserved hypothetical protein	7256	3691	5759	4859	1.3	0.208	2.0	0.000	1.5	0.011
MGG_08203	multiprotein-bridging factor 1	3	3	3	3	1.0	0.656	1.0	0.156	1.0	0.152
MGG_08232	LPS glycosyltransferase	45897	29976	16424	23345	2.8	0.062	1.5	0.260	2.0	0.078
MGG_08304	mechanosensitive ion channel family	38	67	32	25	1.2	0.455	0.6	0.017	1.5	0.181

MGG_08370	1,3-beta-glucanosyltransferase gel3	76341	78496	53726	47494	1.4	0.085	1.0	0.861	1.6	0.026
MGG_08435	predicted protein	143	152	195	130	0.7	0.218	0.9	0.851	1.1	0.611
MGG_08449	related to tol protein	84	32	34	29	2.5	0.002	2.7	0.000	2.9	0.001
MGG_08487	cellobiose dehydrogenase	1833	551	449	568	4.1	0.084	3.3	0.034	3.2	0.027
MGG_08489	predicted protein	4	4	4	4	1.0	0.143	1.0	0.810	1.0	0.050
MGG_08542	small s protein	207	88	198	59	1.0	0.881	2.4	0.051	3.5	0.014
MGG_08543	conserved hypothetical protein	61204	14154	9894	2451	6.2	0.058	4.3	0.016	25.0	0.004
MGG_08662	predicted protein	59	43	37	42	1.6	0.186	1.4	0.195	1.4	0.162
MGG_08663	conserved hypothetical protein	9674	9256	10262	5391	0.9	0.824	1.0	0.837	1.8	0.001
MGG_08725	D-lactate dehydrogenase	98123	127790	83292	72668	1.2	0.515	0.8	0.096	1.4	0.136
MGG_08809	conserved hypothetical protein	661	510	616	420	1.1	0.689	1.3	0.165	1.6	0.001
MGG_08810	2,5-diketo-D-gluconic acid reductase A	683	597	928	784	0.7	0.451	1.1	0.700	0.9	0.595
MGG_08832	C-5 sterol desaturase	2578	7056	2574	2357	1.0	0.996	0.4	0.037	1.1	0.769
MGG_09079	GPI anchored protein	675	846	1504	3970	0.4	0.243	0.8	0.788	0.2	0.130
MGG_09082	cell wall surface anchor family protein	9387	11398	9926	10381	0.9	0.726	0.8	0.234	0.9	0.442
MGG_09109	WSC domain protein	6	10	6	6	1.0	0.942	0.6	0.071	1.0	0.978
MGG_09110	hypothetical protein	92122	109857	46540	117060	2.0	0.048	0.8	0.288	0.8	0.106
MGG_09125	osmosensor protein	746	786	359	597	2.1	0.006	0.9	0.619	1.2	0.036
MGG_09164	glucan 1,4-alpha-maltohexaosidase precursor	17	20	25	23	0.7	0.106	0.9	0.605	0.8	0.479
MGG_09166	predicted protein	22803	27727	39288	37630	0.6	0.016	0.8	0.404	0.6	0.018
MGG_09211	sulfite reductase flavoprotein alpha-component	3636	5450	4925	2574	0.7	0.114	0.7	0.243	1.4	0.072
MGG_09250	gamma-butyrobetaine dioxygenase	115	120	108	119	1.1	0.721	1.0	0.841	1.0	0.880
MGG_09361	hypothetical protein	4420	2464	2071	956	2.1	0.031	1.8	0.042	4.6	0.003
MGG_09594	predicted protein	12	15	7	19	1.7	0.189	0.8	0.645	0.6	0.232
MGG_09612	predicted protein	2349	1111	3228	1030	0.7	0.414	2.1	0.020	2.3	0.001
MGG_09640	alpha-amylase 1	22849	24296	18050	15868	1.3	0.013	0.9	0.665	1.4	0.000
MGG_09641	extracellular proline-serine rich protein	14437	22204	19388	19689	0.7	0.155	0.7	0.310	0.7	0.090
MGG_09642	alpha-amylase	4613	4943	2215	3024	2.1	0.025	0.9	0.796	1.5	0.071
MGG_09736	extracellular conserved serine-rich protein	182061	216550	148556	162163	1.2	0.407	0.8	0.394	1.1	0.552
MGG_09761	hypothetical protein	31396	39798	82627	38649	0.4	0.001	0.8	0.168	0.8	0.049
MGG_09928	conserved hypothetical protein	5302	3440	3326	4143	1.6	0.040	1.5	0.000	1.3	0.013
MGG_10024	hypothetical protein	1586	759	428	289	3.7	0.021	2.1	0.100	5.5	0.013
MGG_10027	calcium-transporting ATPase 1	1942	3212	1561	2644	1.2	0.332	0.6	0.019	0.7	0.074
MGG_10057	conserved hypothetical protein	47591	16895	15281	12059	3.1	0.003	2.8	0.002	3.9	0.001
MGG_10058	predicted protein	7284	4946	3553	6769	2.1	0.031	1.5	0.081	1.1	0.723
MGG_10059	Gelsolin repeat-containing protein	32321	22257	14009	35191	2.3	0.000	1.5	0.006	0.9	0.644
MGG_10104	zinc metalloprotease mde10 precursor	13341	10757	4781	4511	2.8	0.005	1.2	0.080	3.0	0.001
MGG_10118	ATPase family AAA domain-containing protein 1	39581	24197	8349	37118	4.7	0.007	1.6	0.130	1.1	0.795
MGG_10131	member of major facilitator superfamily multidrug-re	9822	4577	4898	3594	2.0	0.056	2.1	0.027	2.7	0.009
MGG_10236	Pfs, NACHT and Ankyrin domain protein	526	136	172	229	3.1	0.001	3.9	0.000	2.3	0.009
MGG_10267	NAD-dependent deacetylase sirtuin-2	346	161	213	199	1.6	0.270	2.1	0.045	1.7	0.099

MGG_10286	conserved hypothetical protein	1688	1813	2091	1664	0.8	0.043	0.9	0.672	1.0	0.841
MGG_10310	conserved hypothetical protein	2382	1483	3366	611	0.7	0.343	1.6	0.226	3.9	0.011
MGG_10324	localization spliceosome protein	10002	13476	6961	9210	1.4	0.251	0.7	0.388	1.1	0.753
MGG_10325	COP9 signalosome complex subunit 2	667	893	420	873	1.6	0.078	0.7	0.210	0.8	0.241
MGG_10400	glucan 1,3-beta-glucosidase	127	59	69	84	1.8	0.074	2.1	0.014	1.5	0.094
MGG_10401	predicted protein	8159	3927	7121	3191	1.1	0.671	2.1	0.103	2.6	0.037
MGG_10575	conserved hypothetical protein	125	138	160	146	0.8	0.514	0.9	0.771	0.9	0.439
MGG_10583	12-oxophytodienoate reductase 1	23347	18825	67514	36370	0.3	0.064	1.2	0.488	0.6	0.324
MGG_10586	conserved hypothetical protein	25422	50252	21740	59780	1.2	0.522	0.5	0.012	0.4	0.006
MGG_10587	conserved hypothetical protein	2483	4681	1535	8642	1.6	0.269	0.5	0.049	0.3	0.050
MGG_10736	hypothetical protein	434	367	357	232	1.2	0.319	1.2	0.325	1.9	0.015
MGG_10848	predicted protein	1982	962	2705	540	0.7	0.249	2.1	0.040	3.7	0.001
MGG_10854	arginase	10	10	7	9	1.5	0.444	1.1	0.867	1.2	0.727
MGG_10868	conserved hypothetical protein	1027	1064	1277	1565	0.8	0.303	1.0	0.870	0.7	0.085
MGG_10869	MFS drug efflux transporter	9995	4207	3600	627	2.8	0.041	2.4	0.062	15.9	0.004
MGG_11047	DNA repair and recombination protein RAD5C	344	112	136	33	2.5	0.031	3.1	0.011	10.3	0.002
MGG_11135	predicted protein	33	15	27	15	2.2	0.034	1.2	0.525	2.2	0.030
MGG_11161	predicted protein	53	47	73	73	1.1	0.566	0.7	0.446	0.7	0.063
MGG_11178	Rho guanyl nucleotide exchange factor	621	622	296	487	1.0	0.998	2.1	0.001	1.3	0.085
MGG_11255	hypothetical protein	148009	151939	183549	87059	0.8	0.438	1.0	0.917	1.7	0.155
MGG_11284	hypothetical protein	1250	103	149	86	12.1	0.106	8.4	0.210	14.5	0.056
MGG_11388	predicted protein	9	10	7	12	0.9	0.509	1.3	0.259	0.8	0.204
MGG_11454	vacuolar calcium ion transporter	7751	6211	3214	4243	2.4	0.052	1.2	0.386	1.8	0.055
MGG_11487	predicted protein	2265	2254	885	2237	1.0	0.982	2.6	0.020	1.0	0.959
MGG_11497	reduced viability upon starvation protein 167	3473	3324	3198	4172	1.1	0.277	1.0	0.582	0.8	0.228
MGG_11529	hypothetical protein	5381	1163	1800	1169	3.0	0.001	4.6	0.000	4.6	0.000
MGG_11534	HLH transcription factor	125	138	160	146	0.8	0.514	0.9	0.771	0.9	0.439
MGG_11548	conserved hypothetical protein	7	10	9	14	0.8	0.386	0.8	0.751	0.5	0.103
MGG_11549	predicted protein	146747	106084	42468	135008	1.4	0.268	3.5	0.003	1.1	0.634
MGG_11597	glucosamine-fructose-6-phosphate aminotransferas	96014	71898	27721	69710	3.5	0.004	1.3	0.437	1.4	0.164
MGG_11608	laccase-2	25	6	8	7	3.3	0.375	4.4	0.327	3.6	0.354
MGG_11609	predicted protein	9	7	6	12	1.4	0.416	1.4	0.432	0.8	0.548
MGG_11643	chromatin structure-remodeling complex protein RS	1255	1645	1205	1103	1.0	0.842	0.8	0.091	1.1	0.198
MGG_11646	predicted protein	6	8	12	6	0.7	0.156	0.5	0.233	1.0	0.920
MGG_11649	PX domain-containing protein	1328	968	397	609	1.4	0.132	3.3	0.001	2.2	0.004
MGG_12106	predicted protein	214	182	72	126	1.2	0.592	3.0	0.024	1.7	0.050
MGG_12283	predicted protein	17	20	27	31	0.9	0.665	0.7	0.140	0.6	0.081
MGG_12324	predicted protein	99	93	48	96	1.1	0.780	2.1	0.049	1.0	0.888
MGG_12824	predicted protein	25	21	16	31	1.2	0.298	1.6	0.013	0.8	0.137
MGG_12833	predicted protein	4	7	8	5	0.6	0.054	0.6	0.160	0.8	0.341
MGG_12910	predicted protein	49	83	60	109	0.6	0.030	0.8	0.662	0.5	0.004

MGG_12922	phospholipid-transporting ATPase 1	4309	2694	860	981	1.6	0.223	5.0	0.005	4.4	0.002
MGG_12926	PHD and RING finger domain protein	442	500	170	542	0.9	0.615	2.6	0.014	0.8	0.283
MGG_12936	alternative oxidase	254773	302282	220117	237483	0.8	0.446	1.2	0.566	1.1	0.705
MGG_13105	predicted protein	47	49	45	22	1.0	0.930	1.0	0.936	2.1	0.016
MGG_13239	linoleate diol synthase	149	187	183	90	0.8	0.511	0.8	0.480	1.7	0.002
MGG_13328	RING finger membrane protein	669	891	419	627	1.6	0.013	0.8	0.344	1.1	0.547
MGG_13346	predicted protein	953	360	157	103	2.6	0.028	6.1	0.045	9.2	0.004
MGG_13350	C6 zinc finger domain protein	11168	2572	3417	810	4.3	0.005	3.3	0.010	13.8	0.002
MGG_13412	predicted protein	31	37	61	43	0.8	0.440	0.5	0.176	0.7	0.053
MGG_13445	predicted protein	49	41	48	46	1.2	0.539	1.0	0.978	1.1	0.772
MGG_13455	mannan endo-1,6-alpha-mannosidase DCW1	706	840	448	616	0.8	0.378	1.6	0.002	1.1	0.183
MGG_13501	predicted protein	18	23	12	27	0.8	0.114	1.5	0.178	0.6	0.056
MGG_13515	predicted protein	165	68	35	71	2.4	0.004	4.8	0.002	2.3	0.006
MGG_13604	predicted protein	940	1120	1093	1073	0.9	0.682	0.8	0.489	0.9	0.440
MGG_13935	oxysterol binding protein (Osh5)	183	73	61	94	2.5	0.200	3.0	0.165	1.9	0.286
MGG_13944	hypothetical protein	135103	30949	14789	6758	4.4	0.062	9.1	0.014	20.0	0.010
MGG_13963	aromatic and neutral aliphatic amino acid permease	48130	83465	63455	49754	0.6	0.079	0.8	0.189	1.0	0.830
MGG_14010	hypothetical protein	4691	2775	2723	1418	1.7	0.104	1.7	0.068	3.3	0.009
MGG_14061	oxalate decarboxylase oxdC	2623	6633	6769	1203	0.4	0.187	0.4	0.115	2.2	0.024
MGG_14151	conserved hypothetical protein	13	7	26	5	0.5	0.525	1.9	0.042	2.4	0.009
MGG_14205	conserved hypothetical protein	28738	33366	11737	25773	2.4	0.013	0.9	0.491	1.1	0.614
MGG_14270	conserved hypothetical protein	584	146	189	253	4.0	0.102	3.1	0.200	2.3	0.194
MGG_14271	predicted protein	7	6	5	8	1.2	0.218	1.5	0.180	0.8	0.184
MGG_14279	glutamine synthetase	12471	15828	4348	12152	2.9	0.001	0.8	0.105	1.0	0.865
MGG_00777	conserved hypothetical protein										
MGG_01945	predicted protein										
MGG_04629	integral membrane protein										
MGG_06927	conserved hypothetical protein										
MGG_11278	mitochondrion protein										
MGG_11604	conserved hypothetical protein										
MGG_11652	endo-1,3(4)-beta-glucanase										
MGG_11675	predicted protein										
MGG_11883	predicted protein										
MGG_11912	predicted protein										
MGG_11986	predicted protein										
MGG_12143	predicted protein										
MGG_12177	translation initiation factor eIF-2B subunit family protein										
MGG_12178	predicted protein										
MGG_12215	predicted protein										
MGG_12225	predicted protein										
MGG_12260	predicted protein										

MGG_12341	predicted protein										
MGG_12565	predicted protein										
MGG_12621	predicted protein										
MGG_12727	predicted protein										
MGG_12757	predicted protein										
MGG_12791	hypothetical protein										
MGG_12795	predicted protein										
MGG_12796	conserved hypothetical protein										
MGG_12823	predicted protein										
MGG_13107	HPP family protein										
MGG_13206	predicted protein										
MGG_13276	erythrocyte membrane protein 1 (PfEMP1)										
MGG_13326	metalloreductase										
MGG_13456	predicted protein										
MGG_13481	conserved hypothetical protein										
MGG_14051	predicted protein										
MGG_14089	NACHT domain protein										
MGG_14138	predicted protein										
MGG_14150	inositol polyphosphate multikinase										
MGG_14314	retrotransposable element Tf2 protein-like protein										
